# Long-term COVID-19 sequelae by Theta and SARS-CoV-2 variants in a Philippine cohort

**DOI:** 10.3389/fmed.2024.1455729

**Published:** 2024-10-02

**Authors:** Cynthia P. Saloma, Marc Edsel C. Ayes, Paolo S. Taracatac, Meryl Rose Q. Asa

**Affiliations:** ^1^Philippine Genome Center, University of the Philippines Diliman, Quezon City, Metro Manila, Philippines; ^2^National Institute of Molecular Biology and Biotechnology, College of Science, University of the Philippines Diliman, Quezon City, Metro Manila, Philippines

**Keywords:** SARS-CoV-2, COVID-19, variants, Long COVID, genomic biosurveillance

## Abstract

**Introduction:**

Millions have been infected with Severe Acute Respiratory Syndrome Coronavirus 2 (SARS-CoV-2) since its emergence in 2019, but most patients make a full recovery. The long-term consequences of the infection are anticipated to unravel in the succeeding years with reports of patients experiencing chronic, debilitating sequelae post-infection commonly referred to as Long COVID. Various Variants of Concern (VoCs) have emerged as the SARS-CoV-2 virus evolved displaying increased infectivity and immune evasiveness. We investigate whether the infecting VoCs affect the sequelae of Long COVID in a Philippine cohort.

**Methods:**

SARS-CoV-2 cases confirmed using RT-PCR followed by Next Generation Sequencing were identified from selected regions of the Philippines and recruited through a retrospective-prospective cohort design. Participants were divided based on the initial infecting VoC or Variant of Interest (VoI) and were subsequently interviewed regarding the presence, intensity, and frequency of key Long COVID symptoms, and followed up on two more separate sessions at least three (3) months apart for a total of three (3) data collection points (S1, S2, S3) to document changes in symptoms throughout the year-long study period.

**Results:**

Long COVID symptoms were reported in 88, 82, and 68% of participants in S1, S2, and S3, respectively, showing declining incidence with elapsed time since the first reported infection. General symptoms including headache, fatigue, and post-exertional malaise were the most frequently reported symptoms, while neuropsychiatric symptoms were the second most frequently reported symptoms. In all three (3) sessions, intermittent brain fog, fatigue, and headache were the most frequently reported symptoms in all SARS-CoV-2 variant cohorts. Factors such as age, sex, comorbidities, and disease severity influenced symptom frequency, providing insight into the risk factors that contribute to the prevalence of this disease.

**Conclusion:**

A large proportion (>68%) of cases in this Philippine cohort previously infected with different SARS-CoV-2 variants presented with long-term complications of COVID-19 characterized by a highly heterogeneous set of debilitating symptoms. The study highlights the need for long-term monitoring of Long COVID and its impact on human health and the need for our health systems to adopt policy response strategies.

## 1 Introduction

In March 2020, the disease caused by Severe Acute Respiratory Syndrome Coronavirus 2 (SARS-CoV-2), also known as Coronavirus disease (COVID-19), spread globally and quickly evolved into a worldwide health crisis that resulted in over 774 million infections and over 7 million deaths across 188 countries and 25 territories worldwide as of February 2024 (1). Apart from the original strain of SARS-CoV-2, variants of the virus bearing fitness-enhancing mutations began to emerge due to evolution and changes in the viral genome, which has implications on infectivity, clinical severity, and diagnostic accuracy (2–4).

Although most vaccinated individuals who contract COVID-19 go on to make a full recovery, there are many reports of patients experiencing chronic, debilitating sequelae post-infection – a phenomenon referred to variably as “Long COVID,” “Long Haulers,” or “Post COVID Syndrome” (5). As a multi-organ system illness, Long COVID encompasses a diverse range of symptoms that can persist for weeks, months, or even years beyond the acute phase of infection. Common manifestations include fatigue, body malaise, peripheral neuropathy or “pins-and-needles” sensation, tinnitus, dyspnea, muscle pain, joint pain or arthritis, gastrointestinal complications, insomnia, cognitive dysfunction, and mood disorders such as depression and anxiety (5, 6). Such symptoms can vary in intensity and frequency as some can be continuous or relapsing and remitting in nature (7). The highly heterogeneous nature of Long COVID underscores the complexity of its pathophysiology, which likely involves a combination of multiple factors such as viral persistence, dysregulated immune responses, neuroinflammation, complications related to comorbidities, and adverse effects of medications used (8).

**Figure 1:**
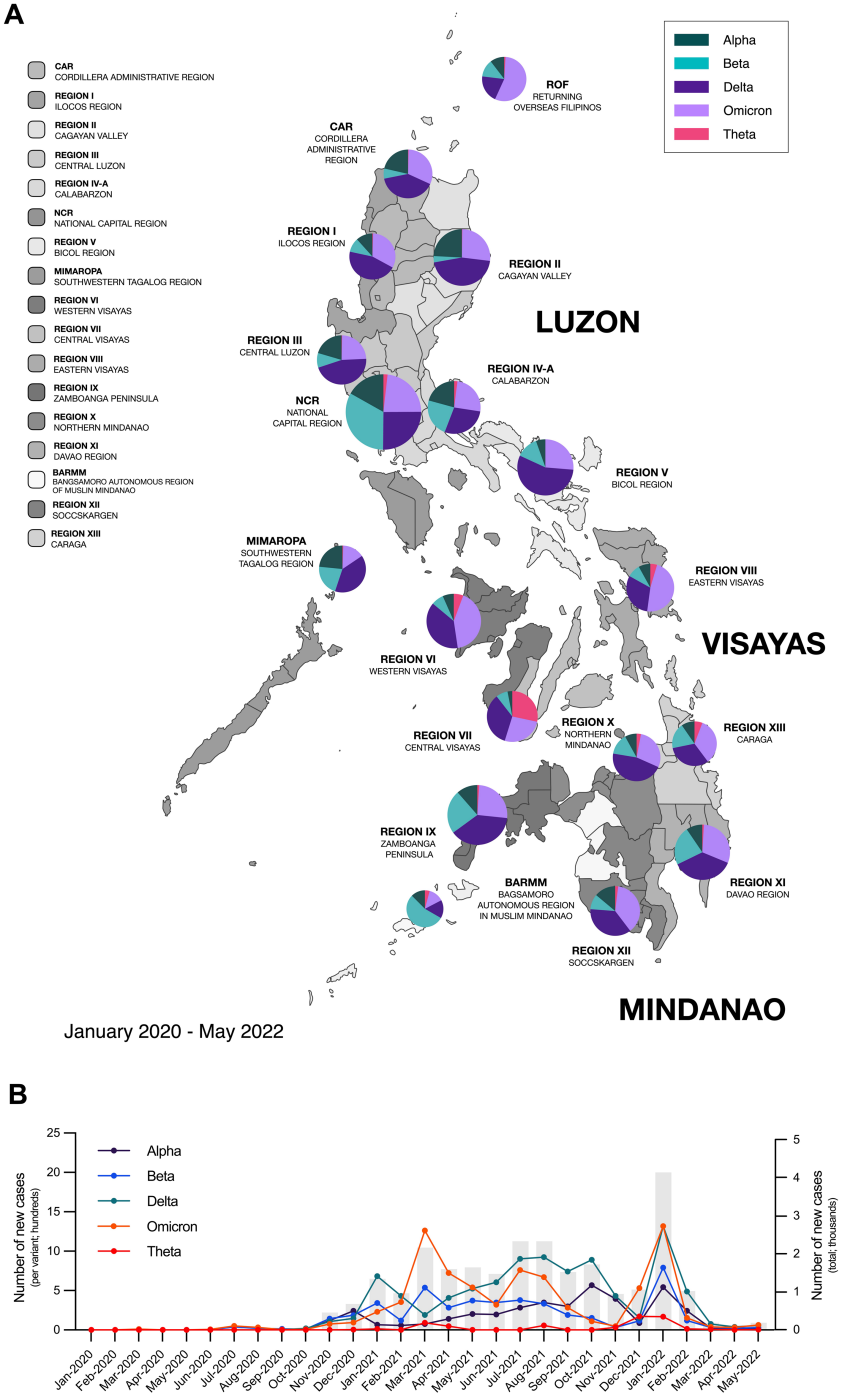
Different SARS-CoV-2 variants circulated throughout the Philippines from 2020–2022. **(A)** Map of the Philippines showing the distribution of circulating SARS-CoV-2 variants from January 2020 to May 2022 across the different regions in the country. **(B)** Epidemiological curve showing the number of new SARS-CoV-2 cases monthly in the Philippines and the incidence of different SARS-CoV-2 variants from January 2020 to May 2022. Bars represent the total number of new cases; data points on the line graph represent the number of new cases per variant; while pie charts show the percentage of circulating variants per region. Epidemiological data was provided by the Core Facility for Bioinformatics of the Philippine Genome Center.

Recent studies have shown that different SARS-CoV-2 variants may influence the clinical course and outcomes of COVID-19, which suggests that different variants may also have implications on the development of Long COVID (9, 10). While the impact of these variants on the severity of acute COVID-19 illness has been extensively studied, their association with Long COVID remains unclear (11). However, emerging evidence suggests that certain variants may be associated with long-term sequelae and distinct clinical phenotypes and patterns which thus raises concerns for the implications on clinical management and post-COVID recovery (10). This was of notable concern at the height of the pandemic when a newly identified Variant of Concern (VoC), dubbed “Theta” variant (P.3) was first identified and described in the Philippines (Figures 1A,B) (12–14).

Despite the growing recognition of Long COVID as a pressing public health concern, there is limited knowledge regarding the pathophysiology of this condition, especially across diverse geographic regions, particularly low- and middle-income countries. In addition, clinical definitions of the diagnosis and management of Long COVID vary between clinical reports since the pathophysiology of the disease is not well-defined, and specific treatments for the disease have yet to be reviewed (6). Thus, this study aims to document, profile, and compare the long-term sequelae of individuals previously infected with confirmed SARS-CoV-2 VoCs (Alpha, Beta, Delta, and Omicron) and the Theta variant in a Philippine cohort. Characterization of variant-specific long-term sequelae post-infection will help enhance post-COVID patient care by providing additional clinical insights for the effective identification of Long COVID and the holistic recovery of affected individuals.

## 2 Methods

### 2.1 Study population and sample

#### 2.1.1 Inclusion criteria

The inclusion criteria for participant selection in the study encompassed individuals meeting the following criteria: aged 15 years and above; residing in the regions of Cebu, Metro Manila, Central Luzon, and West Visayas; confirmed to have tested positive for SARS-CoV-2 infection, specifically either the Theta variant or any other VoC; and whose samples were included in the Department of Health-Epidemiology Bureau (DOH-EB) COVID-19 Biosurveillance Program.

#### 2.1.2 Sample collection and processing

Samples from the Department of Health Epidemiology Bureau (DOH-EB) SARS-CoV-2 Biosurveillance Program were collected as nasopharyngeal swabs from patients undergoing routine COVID-19 testing at any of the over 200 accredited testing centers across the country. The nasopharyngeal swabs were stored in refrigerated temperatures (4°C) pending transport to any of the 17 regional collection centers throughout the Philippine archipelago, after which the samples were forwarded to the University of the Philippines Philippine Genome Center in Quezon City under dry ice following a hub-and-spoke laboratory network setup. All samples were pre-screened and re-tested using SARS-CoV-2 RT PCR prior to whole genome sequencing following Illumina’s COVIDSeq target enrichment protocol. Samples were deemed adequate for sequencing if the resulting Ct value in RT-PCR was lower than 30 in all gene targets. Average genome coverage of all samples sequenced either with NovaSeq 6,000, NextSeq 5,000 or MiSeq system of Illumina with this inclusion criteria was 98.93% (IQR 93.76–99.88).

#### 2.1.3 Genomic sequencing and variant determination

SARS-CoV-2 whole genome sequencing (WGS) was performed at the DNA Sequencing Core Facility of the University of the Philippines Philippine Genome Center (PGC-DSCF). Manual RNA extraction using QIAamp viral RNA mini kit (3) or automated RNA extraction was performed either via the Magabio Plus Virus DNA/RNA Purification Kit or the 3DMed 96A Automated Viral RNA Purification Kit using the Thermo Scientific TMKingFisher TMFlex Purification System. Prior to library preparation, all samples were confirmed to be SARS-CoV-2 positive via RT-PCR as a quality control measure for viral gRNA integrity as evaluated by a clinical pathologist.

SARS-CoV-2 WGS performed at the PGC-DSCF followed COVIDSeq protocol of Illumina (Document # 1000000128490, version 3-January 2022) that was previously optimized by the DSCF team for use with the Illumina COVIDSeq Test (RUO) Kit (Part number 20043675) as previously described for the first detection of the B.1.1.7 variant in the Philippines (3) and the Theta variant (14). NovaSeq6000 was used to sequence samples in batches of 750 while fewer samples were run on NextSeq500 or MiSeq sequencing platforms.

Reference-based assembly, variant calling and lineage assignment were as previously described (14) and performed by the bioinformatics team at the UP PGC Core Facility for bioinformatics. Briefly, sequence reads were mapped to the reference SARS-CoV-2 genome sequence (NCBI accession no. NC_045512.2) using minimap2 v2.17 (15) and further processed using Samtools v1.10 (16) with consensus sequence generation and variant calling done using iVar v1.3 (17). SARS-CoV-2 lineages were assigned using pangolin v4.1.3 and its subsequent updates (18) and the tools MUMmer v4.0 (19) and RATT were used for variant annotation (20). SARS-CoV-2 phylogenetic trees were generated using CFB’s local instance of the Nextstrain analysis platform (21). As of the end of July 2024, a total of 56,571 SARS-CoV-2 samples have been sequenced locally of which 3,737, 4,486, 8,800 and 608 were classified as alpha, Beta, Delta, and Theta variants, respectively.

#### 2.1.4 Patient recruitment

**Figure 2:**
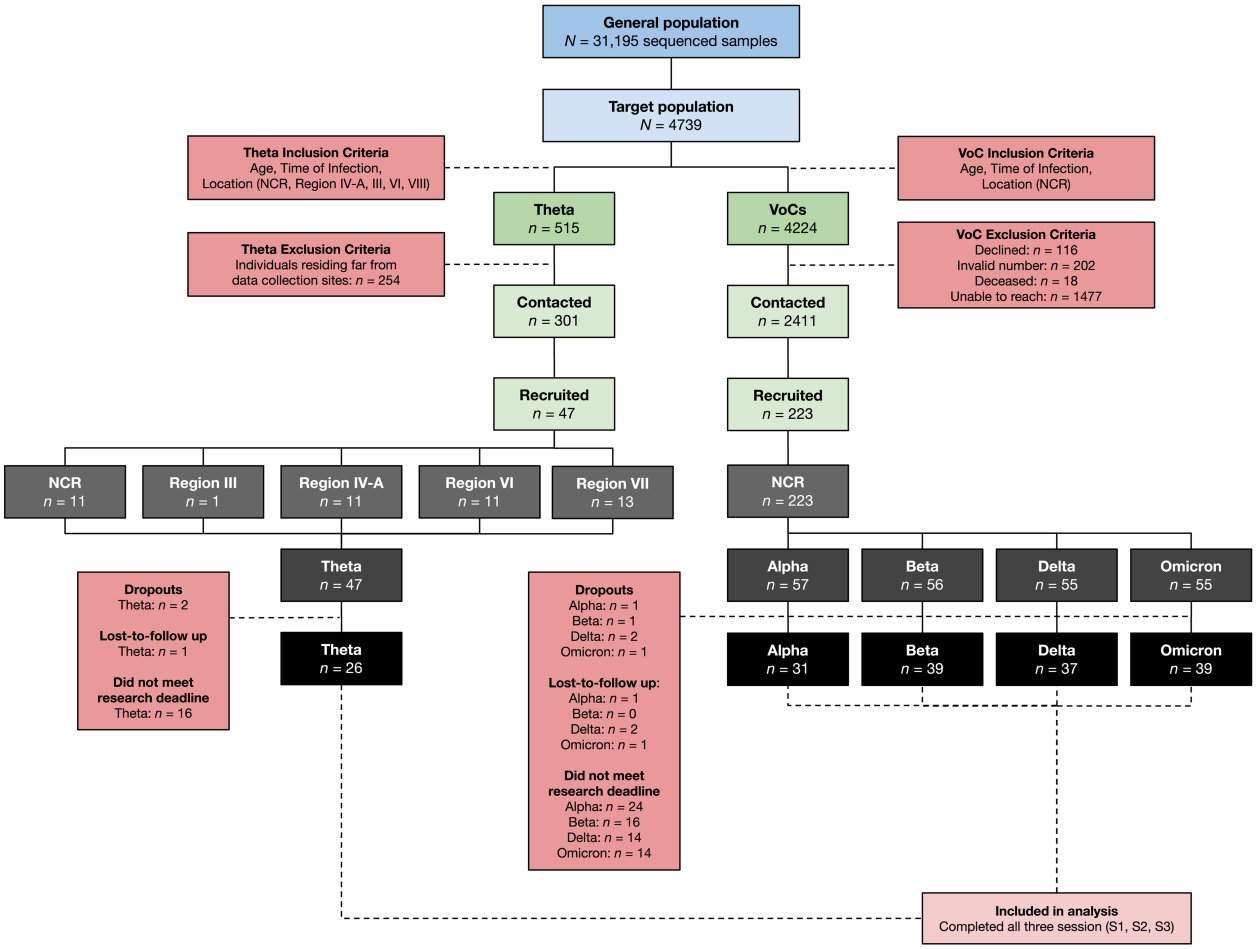
Research participant selection. SARS-CoV-2 cases from the NCR, Region III, Region IV-A, Region VI, and Region VII confirmed using Next Generation Sequencing from January 2021 to January 2022 were recruited into the study using an inclusion criteria based on location, time of infection, and age. Only data from those who completed the initial interview (S1) and both follow-up interviews (S2, S3) were included in the final analysis.

Samples processed by the Philippine Genome Center between January 2021 to January 2022 were reviewed for eligibility with 4,739 potential respondents identified. From this pool, patients were selected via convenience sampling to be contacted for recruitment and interview after first being submitted to the DOH-EB for deanonymization (Figure 2). After the initial interview, participants were scheduled for two more separate sessions at a minimum three-month interval for a total of three data collection points (S1, S2, and S3).

From the pool of potential respondents, 460 patients were confirmed cases of COVID-19 due to the Theta variant. Initial sample size calculation yielded a target sample size of 134 patients per VoC cohort following assumptions of increased hospitalization rates in VoCs compared to non-VoCs of 20 and 7.5%, respectively, (22). Difficulties in establishing contact with historical cases and loss-to-follow up resulted in a final case recruitment rate of only 10% in the Theta variant cohort, yielding only 47 cases of Theta who ultimately agreed to enroll in the study. This was matched with at least 50 cases from each respective WHO-VoC cohort, resulting in a final recruitment of cases of Alpha (*n* = 57), Beta (*n* = 56), Delta (*n* = 55), Omicron (*n* = 55), and Theta (*n* = 47).

The following regions represent some of the main geographic locations where Theta was detected in the Philippines: The National Capital Region (NCR) – of which includes Metro Manila – Region IV-A, Region VI, and Region VII. Although Region III only accounted for 2% of the total Theta cases in the country, it was still considered in the study due to its proximity to the NCR. Within each region, key cities and provinces with the highest number of reported Theta cases were identified for recruitment into the study. Overall, 47 participants were recruited for the Theta variant: 13 from Region VII, 11 from Region VI, 11 from the NCR, 11 from Region IV-A, and one from Region III; while 223 participants were recruited for VoCs, all of which were from the NCR. Although a total of 270 participants were successfully recruited, not all were able to complete both follow-up interviews by the time of manuscript preparation, resulting in a smaller sample size for Alpha (*n* = 31), Beta (*n* = 39), Delta (*n* = 37), Omicron (*n* = 39), and Theta (*n* = 26).

### 2.2 Interview and data collection

Interviews were conducted at least one (1) year and eight (8) months from the time of infection, and interview questions focused on identifying and qualifying the presence, intensity, and frequency of long-term sequelae related to Long COVID based on the clinical definitions established through a Delphi consensus led by the World Health Organization (WHO) (23). Responses to the interview questions were recorded in a data collection form.

### 2.3 Ethics approval

As a multi-site study, ethics approval for the study was obtained from the Single Joint Research Ethics Board of the Department of Health (DOH-SJREB), University of the Philippines Manila Research Ethics Board (UPMREB/NCR), Vicente Sotto Memorial Medical Center Research Ethics Committee (VSMMC-REC/Region VII), Teresita L. Jalandoni Provincial Hospital Ethics Review Committee (TLJPH-ERC/Region VI), and West Visayas State University Ethics Review Committee (WVSU-ERC/Region VI) institutional review boards. The study was conducted in accordance with the local legislation and institutional requirements, and participants provided their written informed consent to participate in this study.

### 2.4 Statistical analysis

Categorical variables were described as frequencies and percentages, while continuous variables were described using the median and mean ± SEM. Categorical variables were compared using a Chi-squared test, while continuous variables were analyzed using parametric tests such as Paired *t-*test and one-way analysis of variance (ANOVA) followed by Tukey’s multiple comparisons test; and non-parametric tests such as Spearman’s rank-order correlation, Mann–Whitney test, and Kruskal–Wallis test followed by Dunn’s multiple comparisons test. All graphs were plotted using GraphPad Prism version 9.0 (GraphPad Software, La Jolla, CA, United States, https://www.graphpad.com/), R version 4.3.3, and RStudio software. All statistical analysis was done using GraphPad Prism version 9.0, and *p* < 0.05 was accepted as statistically significant in both the main analyses and *post hoc* tests.

## 3 Results

### 3.1 Baseline characteristics of SARS-CoV-2 cases recruited into the study

Baseline characteristics refer to the characteristics of participants recruited into the study and are summarized in Tables 1–3. The median age of each SARS-CoV-2 variant cohort was 28–35 years old. Across variant cohorts, the majority of Alpha (18 [58.1%]) and Delta (15 [40.5%]) cases were ages 20–29, while the majority of Beta (15 [38.5%]), Omicron (20 [51.3%]), and Theta (11 [42.3%]) cases belonged to ages 30–39. In terms of sex, the majority of Alpha (18 [58.1%]) cases were male, while the majority of Beta (23 [59.0%]), Delta (19 [51.4%]), Omicron (23 [59.0%]), and Theta (16 [61.5%]) cases were female. In terms of comorbidities, an equal number of participants (86 [50.0%]) reported having comorbidities or no comorbidities. Among those that did have underlying comorbidities, the majority reported having hypertension (20 [11.6%]), diabetes (19 [11.0%]), and allergies (31 [18.0%]) (Table 1). It is notable that the Philippines has the highest number of sequenced Beta cases at 4486 cases at the end of 2021 (Figure 1).

**Table 1:** Demographics of SARS-CoV-2 cases recruited into the study.

Characteristic	Alpha (*N* = 31)	Beta (*N* = 39)	Delta (*N* = 37)	Omicron (*N* = 39)	Theta (*N* = 26)	*P-*value^†^
Age (years)						
Median (IQR)	28 (24–37)	33 (27–42)	31 (26–40)	35 (30–41)	31 (26–39)	0.2177
Age group, *n* (%)						
10–19	1 (3.2)	2 (5.1)	1 (2.7)	0 (0)	0 (0)	0.0858
20–29	18 (58.1)	10 (25.6)	15 (40.5)	8 (20.5)	10 (38.5)	
30–39	6 (19.4)	15 (38.5)	12 (32.4)	20 (51.3)	11 (42.3)	
40–49	3 (9.7)	8 (20.5)	2 (5.4)	9 (23.1)	4 (15.4)	
50–59	3 (9.7)	2 (5.1)	5 (13.5)	1 (2.6)	1 (3.8)	
60–69	0 (0)	2 (5.1)	2 (5.4)	1 (2.6)	0 (0)	
Sex, *n* (%)						
Male	18 (58.1)	16 (41.0)	18 (48.6)	16 (41.0)	10 (38.5)	0.5136
Female	13 (41.9)	23 (59.0)	19 (51.4)	23 (59.0)	16 (61.5)	
Number of comorbidities, *n* (%)						
Zero (0)	16 (51.6)	19 (48.7)	13 (35.1)	20 (51.3)	18 (69.2)	0.7020
One (1)	9 (29.0)	12 (30.8)	14 (37.8)	11 (28.2)	6 (23.1)	
Two (2)	4 (12.9)	6 (15.4)	6 (16.2)	6 (15.4)	2 (7.7)	
Three (3)	2 (6.5)	1 (2.6)	1 (2.7)	2 (5.1)	0 (0)	
Four (4)	0 (0)	1 (2.6)	3 (8.1)	0 (0)	0 (0)	
Comorbidities, *n* (%)						
Hypertension	4 (12.9)	6 (15.4)	5 (13.5)	5 (12.8)	0 (0)	0.7340
Diabetes	3 (9.7)	3 (7.7)	7 (18.9)	5 (12.8)	1 (3.8)	
Heart disease	0 (0)	1 (2.6)	1 (2.7)	2 (5.1)	1 (3.8)	
Lung disease	1 (3.2)	0 (0)	1 (2.7)	0 (0)	0 (0)	
Gastrointestinal	1 (3.2)	1 (2.6)	0 (0)	1 (2.6)	1 (3.8)	
Genito-urinary	0 (0)	1 (2.6)	0 (0)	1 (2.6)	0 (0)	
Neurological	0 (0)	0 (0)	2 (5.4)	0 (0)	1 (3.8)	
Cancer	1 (3.2)	1 (2.6)	0 (0)	2 (5.1)	1 (3.8)	
Allergies	6 (19.4)	10 (25.6)	10 (5.8)	4 (10.3)	1 (3.8)	
Asthma	3 (7.0)	2 (5.4)	5 (12.7)	6 (10.9)	0 (0)	
Skin disease	1 (3.5)	1 (1.8)	2 (5.5)	0 (0)	0 (0)	
Others	3 (9.7)	5 (12.8)	5 (13.5)	3 (7.7)	4 (15.4)	

^†^Statistically significant associations and differences were determined through Chi-Square test and Kruskal–Wallis test followed by Dunn’s multiple comparisons test, respectively. After applying multiple corrections, the threshold for statistical significance was adjusted to *P* < 0.05 (^*^P < 0.05).

**Table 2:** Vaccination profile of SARS-CoV-2 cases recruited into the study.

Characteristic	Alpha (*N* = 31)	Beta (*N* = 39)	Delta (*N* = 37)	Omicron (*N* = 39)	Theta (*N* = 26)	*P-*value^†^
Vaccination status, *n* (%)						
Unvaccinated	0 (0)	1 (2.6)	0 (0)	0 (0)	1 (3.8)	0.5268
Complete primary series	5 (16.1)	4 (10.3)	4 (10.8)	1 (2.6)	3 (11.5)	
One booster shot	14 (45.2)	16 (41.0)	16 (43.2)	15 (38.5)	14 (53.8)	
Two booster shots	12 (38.7)	18 (46.2)	17 (45.9)	23 (59.0)	8 (30.8)	
Vaccine combination, *n* (%)						
Homologous	8 (25.8)	14 (35.9)	10 (27.0)	3 (7.7)	9 (34.6)	**0.0333**
Heterologous	23 (74.2)	24 (61.5)	27 (73.0)	36 (92.3)	16 (61.5)	
Vaccine type, *n* (%)						
Inactivated whole virus	2 (6.5)	0 (0)	3 (8.1)	0 (0)	3 (11.5)	**0.0294**
mRNA	4 (12.9)	14 (35.9)	6 (16.2)	2 (5.1)	6 (23.1)	
Viral vector	2 (6.5)	0 (0)	1 (2.7)	1 (2.6)	0 (0)	
Inactivated whole virus + mRNA	10 (32.3)	15 (38.5)	15 (40.5)	15 (38.5)	9 (34.6)	
Inactivated whole virus + viral vector	0 (0)	2 (5.1)	2 (5.4)	5 (12.8)	2 (7.7)	
mRNA + Viral vector	13 (41.9)	7 (17.9)	10 (27.0)	16 (41.0)	5 (19.2)	
Time elapsed since most recent vaccination (days), median (IQR)						
Time elapsed since vaccination and S1	390 (355–425)	390 (338–425)	387 (345–418)	376 (347–425)	354 (292–457)	0.9722
Time elapsed since vaccination and S2	478 (446–516)	481 (439–523)	481 (444–509)	474 (439–523)	460 (386–571)	0.9952
Time elapsed since vaccination and S3	565 (537–597)	591 (541–624)	570 (530–602)	565 (530–633)	561 (510–677)	0.8746
Time elapsed between pre-infection vaccination date and infection (days), median (IQR)	17 (13–49)	37 (5–69)	64 (39–109)	59 (33–167)	23 (13–101)	0.2124
Time elapsed between infection and post-infection vaccination date (days), median (IQR)	161 (111–222)	194 (140–238)	125 (74–151)	104 (69–223)	131 (90–208)	0.1846

^†^Statistically significant associations and differences were determined through Chi-Square test and Kruskal–Wallis test followed by Dunn’s multiple comparisons test, respectively. After applying multiple corrections, the threshold for statistical significance was adjusted to *P* < 0.05 (^*^P < 0.05). Bold values indicate statistically significant *P*  values.

**Table 3:** Infection profile of SARS-CoV-2 cases recruited into the study.

Characteristic	Alpha (*N* = 31)	Beta (*N* = 39)	Delta (*N* = 37)	Omicron (*N* = 39)	Theta (*N* = 26)	*P-*value^†^
Total number infections						
One (1)	19 (61.3)	28 (71.8)	22 (59.5)	17 (43.6)	24 (92.3)	**0.0003**
Two (2)	11 (35.5)	10 (25.6)	15 (40.5)	15 (38.5)	2 (7.7)	
Three (3)	1 (3.2)	1 (2.6)	0 (0)	7 (17.9)	0 (0)	
Disease severity during acute infection						
Asymptomatic	2 (6.5)	1 (2.6)	3 (8.1)	2 (5.1)	1 (3.8)	0.5548
Mild	26 (83.9)	33 (84.6)	25 (67.6)	34 (87.2)	21 (80.8)	
Moderate	3 (9.7)	3 (7.7)	9 (24.3)	3 (7.7)	4 (15.4)	
Severe	0 (0)	2 (5.1)	0 (0)	0 (0)	0 (0)	
Time elapsed since infection (days), median (IQR)						
Time elapsed since infection and S1	736 (701–771)	736 (684–771)	733 (691–763)	722 (693–771)	700 (637–803)	0.9722
Time elapsed since infection and S2	824 (792–862)	827 (785–869)	827 (790–855)	820 (785–869)	806 (732–917)	0.9952
Time elapsed since infection and S3	911 (883–943)	937 (887–970)	916 (876–948)	911 (876–979)	908 (856–1,024)	0.8732

^†^Statistically significant associations and differences were determined through Chi-Square test and Kruskal–Wallis test followed by Dunn’s multiple comparisons test, respectively. After applying multiple corrections, the threshold for statistical significance was adjusted to *P* < 0.05 (^***^P < 0.001). Bold values indicate statistically significant *P*  values.

The Philippines utilized a highly heterogeneous set of primary SARS-CoV-2 vaccines during the pandemic, with a very high vaccination rate (>96% of the target population) in the National Capital Region (NCR), the main site of our study (24). In terms of vaccination status upon recruitment into the study, only two (1.16%) were unvaccinated while the majority of cases had at least one booster shot (153 [89.0%]). In particular, the majority of Alpha (14 [45.2%]) and Theta (14 [53.8%]) cases have had one booster shot, while the majority of Beta (18 [46.2%]), Delta (17 [45.9%]), and Omicron (23 [59.0%]) cases have had two booster shots. Across variant cohorts, the majority of cases took a heterologous vaccine combination (126 [73.3%]), with the remaining cases taking a homologous vaccine combination (44 [25.6%]). Among those who took a homologous vaccine combination, the majority of cases received an mRNA vaccine (i.e., Cominarty [Pfizer-BioNTech] and Spikevax [Moderna]) (32 [18.6%]). Among those who took a heterologous vaccine combination, the majority of Alpha (13 [41.9%]) and Omicron (16 [41.0%]), cases received a combination of an mRNA and viral vector vaccine, while the majority of Beta (15 [38.5%]), Delta (15 [40.5%]), and Theta (9 [34.6%]) cases took a combination of an inactivated whole virus and mRNA vaccine. In terms of the time elapsed since the most recent vaccination for each variant cohort, the median time elapsed prior to S1 ranged from 354 to 390 days; the median time elapsed prior to S2 ranged from 460 to 481 days; while the median time elapsed prior to S3 ranged from 561 to 591 days. Among those who were vaccinated prior to infection, the median time elapsed before infection ranged from 17 to 64 days, which is a period associated with optimal vaccine efficacy. We also accounted for those who were vaccinated post-infection, with the median time elapsed since their infection ranging from 104 to 194 days (Table 2).

In terms of total number of infections, the majority of cases reported having only one confirmed infection (i.e., from the variant of interest) (110 [64.0%]). At the time of infection, the majority of cases reported symptoms regardless of variant (163 [94.8%]). Symptomatic cases were further classified based on disease severity, wherein cases were categorized as mild if individuals experienced symptoms; moderate if they exhibited an oxygen saturation level below 95% and required supplemental oxygen; and severe if they were admitted to the intensive care unit (ICU) and required intubation. The majority of cases were classified as mild (139 [80.8%]), while only 22 (12.8%) and two (1.16%) cases were classified as moderate and severe, respectively. In terms of the time elapsed since infection with the variant of interest, the median time elapsed prior to S1 ranged from 700 to 736 days; the median time elapsed prior to S2 ranged from 806 to 827 days; while the median time elapsed prior to S3 ranged from 908 to 937 days (Table 3).

### 3.2 Long COVID symptoms are prevalent and predominantly fall under general and neuropsychiatric sequelae

**Figure 3:**
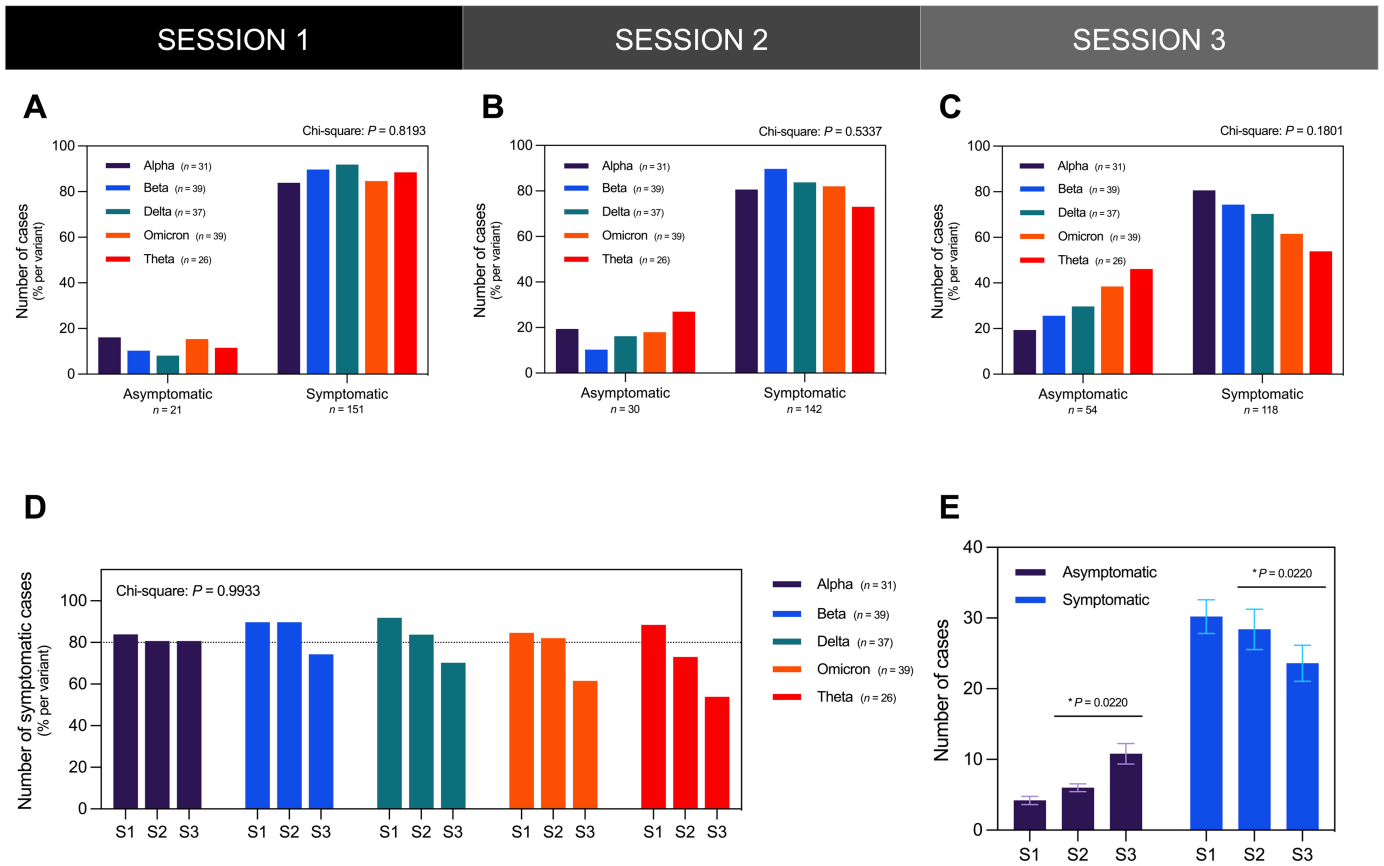
The majority of the participants presented as symptomatic for Long COVID in all three sessions regardless of infecting SARS-CoV-2 variant. The distribution of the number of asymptomatic and symptomatic cases did not vary significantly across different SARS-CoV-2 variants in **(A)** S1 (Chi-Square test; *P* = 0.8193), **(B)** S2 (Chi-Square test; *P* = 0.5337), and **(C)** S3 (Chi-Square test; *P* = 0.1801). **(D)** The distribution of the number of symptomatic cases per variant did not likewise vary significantly during the three interview sessions (Chi-Square test; *P* = 0.9933). **(E)** From interview sessions S2 to S3 which occurred 3 months apart, the number of asymptomatic cases significantly increased while the number of symptomatic cases significantly decreased (Paired *t*-test; *P* = 0.0220). Bars represent the percentage of cases per variant or mean ± SEM with statistically significant associations and differences determined through Chi-Square test and Paired *t*-test, respectively (^*^P < 0.05, ^ns^*P* > 0.05).

In all three sessions, most participants reported experiencing sequelae post-infection regardless of variant (Figures 3A–C). In the first session (S1), Long COVID symptoms were reported in 88% of participants with more than 80% of each variant cohort reporting symptoms (Figure 3A). In the second session (S2) and third session (S3), the number of those reporting symptoms decreased to 82% (Figure 3B) and 68% (Figure 3C) of participants, respectively. From S1 to S2, only the Alpha and Beta cohorts consistently had at least 80% reporting symptoms. However, from S2 to S3, the number of those reporting symptoms remained unchanged in the Alpha cohort while the number decreased in the Beta cohort (Figure 3D). In contrast, the Delta, Omicron, and Theta cohorts exhibited a decrease in the number of those reporting symptoms from S1 to S3, although this was not statistically significant (Figure 3D). Across all variants, there was a significant increase in the number of symptomatic cases concurrent with the observed decrease in the number of asymptomatic cases from S2 to S3 (Figure 3E).

To systematically analyze and understand the breadth of Long COVID symptomatology, seven (7) main categories were identified based on the major organ systems affected by common Long COVID symptoms: general symptoms, cardiopulmonary symptoms, gastrointestinal symptoms, musculoskeletal symptoms, neuropsychiatric symptoms, dermatologic symptoms, and women-related symptoms.

**Figure 4:**
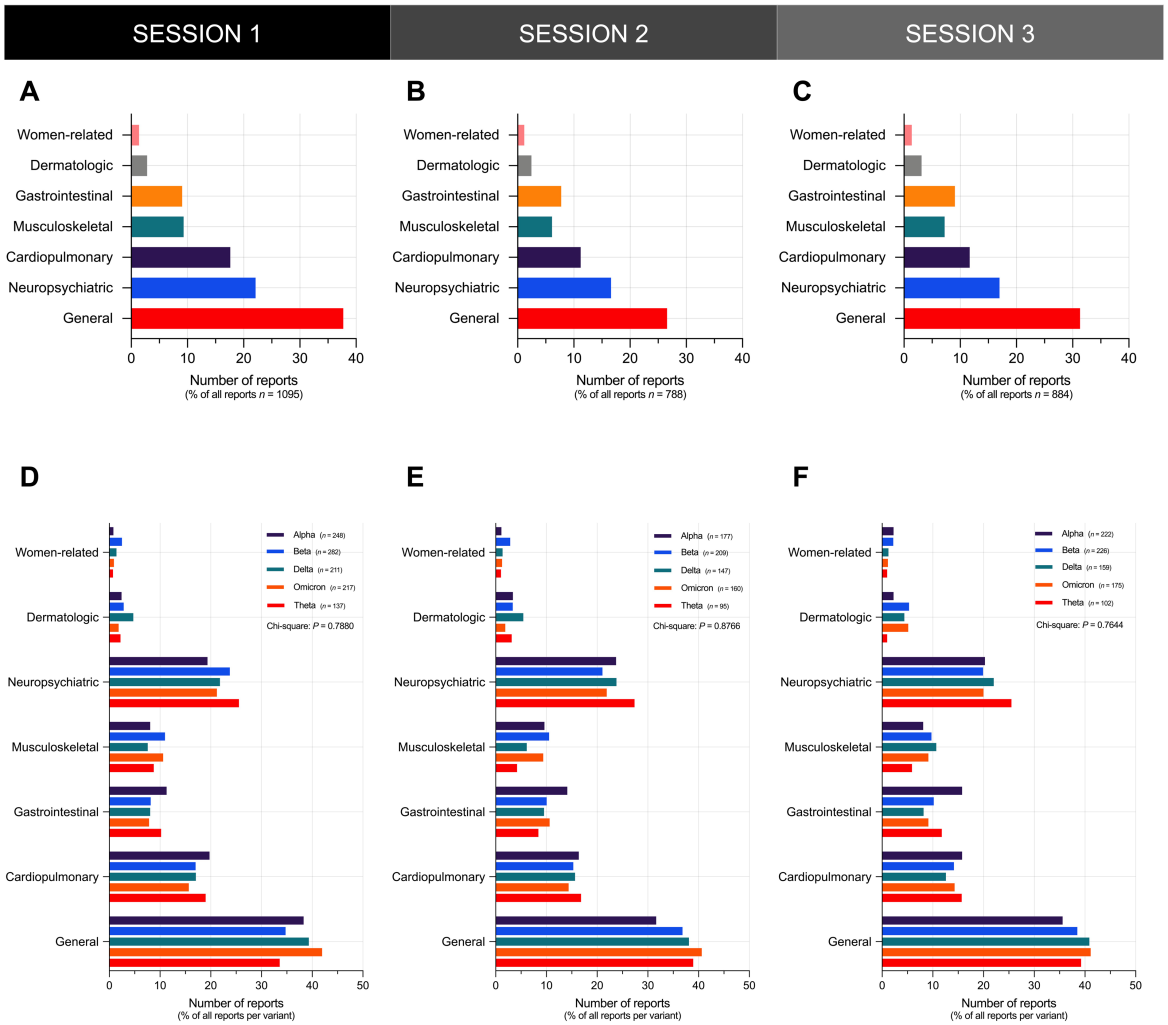
General and neuropsychiatric Long COVID symptoms represent the top two categories of the most frequently reported symptoms. Shown is the overall frequency of Long COVID symptoms reported by participants in **(A)** S1, **(B)** S2, and **(C)** S3. The distribution of the frequency of Long COVID symptoms reported by participants did not vary significantly across different SARS-CoV-2 variants in **(D)** S1 (Chi-Square test; *P* = 0.7880), **(E)** S2 (Chi-Square test; *P* = 0.8766), and **(F)** S3 (Chi-Square test; *P* = 0.7644). Bars represent the percentage of the total number of reports in the sample or the percentage of reports per variant with statistically significant associations determined through Chi-Square test (^*^P < 0.05, ^ns^*P* > 0.05).

In all three sessions, general and neuropsychiatric Long COVID symptoms represented the top two categories of the most frequently reported symptoms across all reports (Figures 4A–C). General symptoms represented the top category of the most frequently reported symptoms across all reports, accounting for over 35% of all reports in S1 (Figure 4A), 25% of all reports in S2 (Figure 4B), and 30% of all reports in S3 (Figure 4C). Neuropsychiatric symptoms represented the second top category of the most frequently reported symptoms across all reports, accounting for over 20% of all reports in S1 (Figure 4A), and over 15% of all reports in S2 (Figure 4B) and S3 (Figure 4C). Upon stratification of participants by variant, general and neuropsychiatric symptoms still represented the top two categories of the most frequently reported symptoms across all reports per variant (Figures 4D–F).

In each of the seven main categories, a comprehensive assessment was conducted to determine the presence of specific Long COVID symptoms in each cohort across the three sessions. General symptoms included headache, dizziness, fever, onset allergies, fatigue, post-exertional malaise, tinnitus, and peripheral neuropathy or “pins-and-needles” sensation; cardiopulmonary symptoms included dyspnea (difficulty breathing), palpitations, cough, and chest pain; gastrointestinal symptoms included stomach pain, diarrhea, constipation, and acid reflux; musculoskeletal symptoms included muscle pain and joint pain; neuropsychiatric symptoms included brain fog (difficulty thinking or concentrating), sleep problems, mood changes, and changes in smell or taste; dermatologic symptoms included rashes and hair loss; and women-related conditions included changes in menstrual cycle.

**Figure 5:**
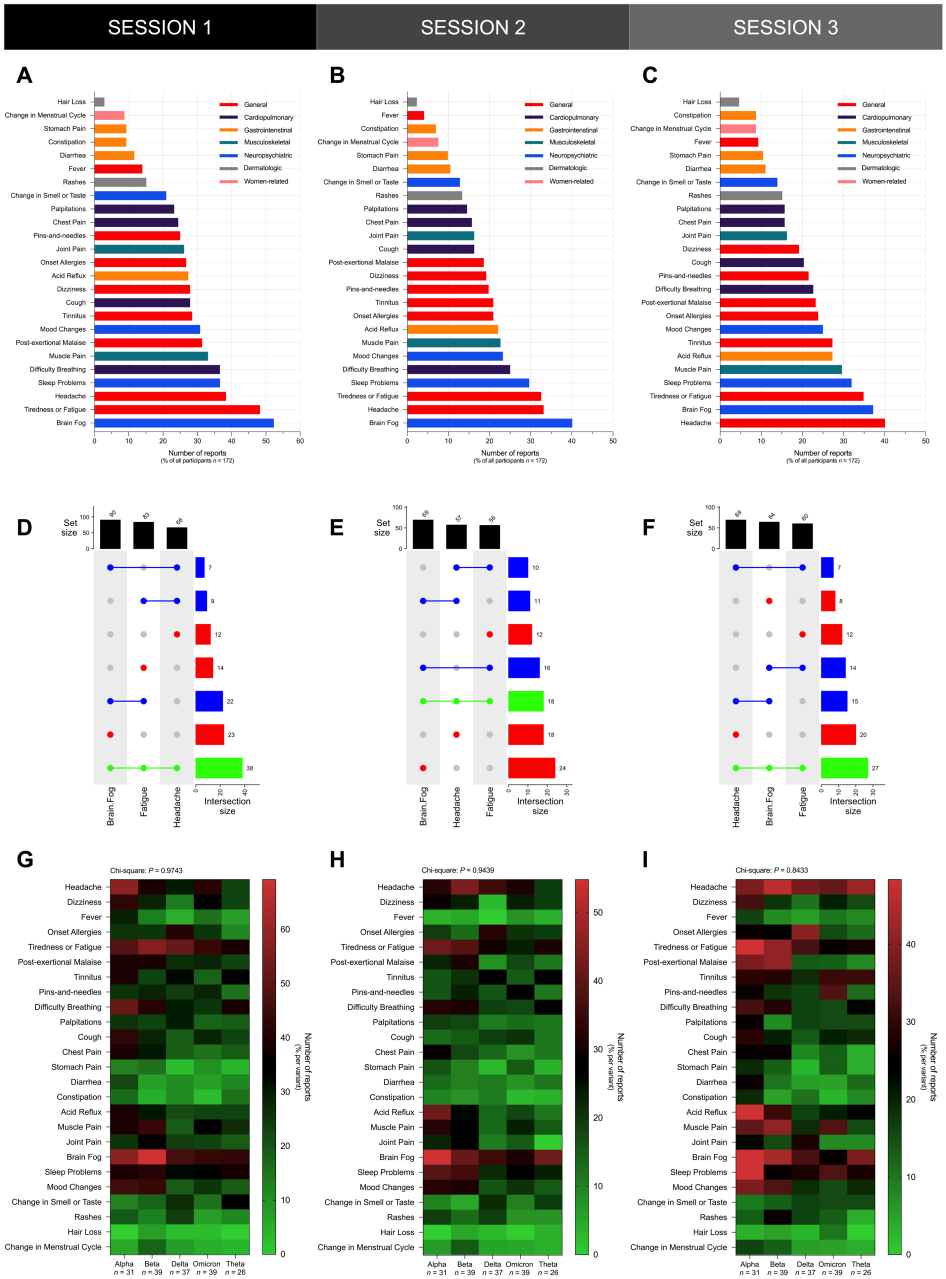
Brain fog, fatigue, and headache were the most frequently reported symptoms across all participants regardless of documented previous infecting SARS-CoV-2 variant. Shown is the overall frequency of specific Long COVID symptoms reported by participants in **(A)** S1, **(B)** S2, and **(C)** S3, and UpSet plots showing the co-occurrence of the top three Long COVID symptoms – namely brain fog, fatigue, and headache – reported by participants in **(D)** S1, **(E)** S2, and **(F)** S3. The distribution of the frequency of specific Long COVID symptoms did not vary significantly across different SARS-CoV-2 variants in **(G)** S1 (Chi-Square test; *P* = 0.9743), **(H)** S2 (Chi-Square test; *P* = 0.9439), and **(I)** S3 (Chi-Square test; *P* = 0.8433). Bars represent the set size, intersection size, or the percentage of all participants in the sample with statistically significant associations determined through the Chi-Square test (^*^P < 0.05, ns *P* > 0.05).

In all three sessions, brain fog, fatigue, and headache, were the most frequently reported symptoms among participants (Figures 5A–C). Brain fog represented the most frequently reported symptom among participants in S1 (Figure 5A) and S2 (Figure 5B), and the second most frequently reported symptom in S3 (Figure 5C). Brain fog was reported in over 50% of participants in S1 (Figure 5A), 40% of participants in S2 (Figure 5B), and over 35% of participants in S3 (Figure 5C). Next to brain fog, fatigue and headache represented the second most frequently reported symptoms in S1 (Figure 5A) and S2 (Figure 5B), respectively, while the latter represented the most frequently reported symptom in S3 (Figure 5C). Fatigue was reported in over 55% of participants in S1 (Figure 5A), while headache was reported in over 35% of participants in S2 (Figure 5B) and S3 (Figure 5C). Notably, brain fog, fatigue, and headache manifested concurrently in more than 22% of participants in S1 (Figure 5D), over 10% of participants in S2 (Figure 5E), and approximately 16% of participants in S3 (Figure 5F).

Upon stratification of participants by variant, the most frequently reported symptoms did not vary significantly across all cohorts in all three sessions since brain fog, headache, and fatigue remained as some of the most frequently reported symptoms in all variant cohorts (Figures 5G–I). In S1, brain fog was the most frequently reported symptom in the Alpha (18 [58%]), Beta (27 [69%]), Omicron (17 [44%]), and Theta (11 [42%]) cohorts, and the second most frequently reported symptom in the Delta (17 [46%]) cohort (Figure 5G). Alongside brain fog, headache was also the most frequently reported symptom in the Alpha (18 [58%]) cohort, and headache was the second most frequently reported symptom in the Omicron (16 [41%]) cohort (Figure 5G). Fatigue was the most frequently reported symptom in the Delta (19 [51%]) cohort, and alongside brain fog, fatigue was also the most frequently reported symptom in the Omicron (17 [44%]) cohort. Fatigue was the second most frequently reported symptom in the Beta (22 [56%]) and Theta (10 [38%]) cohorts (Figure 5G). In S2, brain fog was the most frequently reported symptom in the Alpha (17 [55%]), Delta (13 [35%]), Omicron (12 [31%]), and Theta (11 [42%]) cohorts, and the second most frequently reported symptom in the Beta (16 [41%]) cohort (Figure 5H). Headache was the most frequently reported symptom in the Beta (17 [44%]) cohort, and alongside brain fog, headache was also the most frequently reported symptom in the Delta (13 [35%]) and Omicron (12 [31%]) cohorts (Figure 5H). Next to brain fog, fatigue was the second most frequently reported symptom in the Alpha (13 [42%]) and Theta (8 [31%]) cohorts (Figure 5H). In S3, the most frequently reported symptoms were fatigue and brain fog in the Alpha (15 [48%]) cohort, and headache in the Beta (18 [46%]), Omicron (14 [36%]), and Theta (11 [42%]) cohorts (Figure 5I). The second most reported symptom was headache in the Alpha (12 [39%]) and Delta (14 [38%]) cohorts, and brain fog in the Beta (17 [44%]) and Theta (10 [38%]) cohorts (Figure 5I).

### 3.3 Long COVID symptoms have diverse manifestations and a predominant relapsing pattern

Apart from identifying the presence of Long COVID symptoms across the different cohorts, participants were also asked to characterize each symptom they encountered by providing detailed descriptions of each sequela (Table 4).

**Table 4:** Summary of Long COVID symptom descriptions provided by participants.

Long COVID symptom^†^	Descriptions
General symptoms	
Headache	Bilateral or unilateral; manifests as a migraine, tension headache, or cluster headache
Dizziness	Manifests as vertigo; feeling of swaying or experiencing earthquake-like sensations
Fever	Sensation of heat, chills, sweating; concurrent with flu-like symptoms
Onset allergies	Allergic rhinitis; allergies to seafood, pets, chicken, eggs
Tiredness or fatigue	More prone to tiredness; experienced after climbing a flight of stairs
Post-exertional malaise	Requires extended recovery time after physical exertion; pain in arms, back, legs, and feet after exercising
Tinnitus	Loud bilateral or unilateral ringing/humming which lasts for 5–10 s; occurs at different times of the day; sensation of deafness or loss of hearing
Pins-and-needles (peripheral neuropathy)	Numbness and difficulty moving hands and feet
Cardiopulmonary symptoms	
Difficulty breathing	Manifests as gasping or sharp sensations during breathing; breathing exhibits a whistling sound; experienced after physical exertion; occurs concurrent with continuous cough
Palpitations	Tachycardia experienced after drinking alcohol or carbonated drinks; can manifest as irregular heartbeats; experienced after physical exertion
Cough	More prone to cough; manifests as an irritated throat or through dry cough; concurrent with colds
Chest pain	Sensation of sharp pain or heaviness pressing on the chest; occurs concurrent with panic attacks; experienced after physical exertion or while resting
Gastrointestinal symptoms	
Stomach pain	Cramping or aching; experienced after drinking caffeine or carbonated drinks
Diarrhea	Bowel movement is usually fast and more frequent; stool is usually soft, and may not necessarily be watery
Constipation	Feeling of strain or pain when passing stools
Acid reflux	Manifests as Gastroesophageal Reflux Disease (GERD); experienced after drinking caffeine or carbonated drinks
Musculoskeletal symptoms	
Muscle pain	Pain in nape, arms, shoulders, upper back, lower back, or legs; experienced after physical exertion
Joint pain	Pain in shoulders, elbows, knees, ankles, and lower back; reported alongside high uric acid and/or cholesterol
Neuropsychiatric symptoms	
Brain fog	Struggle with maintaining focus over extended periods, recalling words, and remembering immediate tasks; memory lapses and periods of dissociation; disorientation resulting in altered motor function
Sleep problems	Difficulty falling asleep; sleep is interrupted and fragmented; concurrent with difficulty breathing and nightmares
Mood changes	More irritable, anxious, depressed, or emotional; less interested in activities
Changes in smell	Manifests as hyposmia, hyperosmia, parosmia, or phantosmia; detection of odors resembling metal, burning, or unpleasant scents
Changes in taste	Manifests as hypogeusia or dysgeusia; more/less sensitive to salty flavors; certain foods lack their expected taste; concurrent with loss of appetite
Dermatologic symptoms	
Rashes	Manifests as atopic dermatitis, urticaria, or Pityriasis rosea; skin is more sensitive and easily irritable; rashes in extremities, face, scalp, or back; concurrent with allergies
Hairfall	Concurrent with itchy scalp
Women-related symptoms	
Changes in menstrual cycle	Shorter or irregular menstrual cycles; decreased menstrual flow; blood appears clumped

^†^List of Long COVID symptoms was based on the clinical definitions of the WHO (23).

A separate assessment was made to characterize the intensity and frequency of Long COVID symptoms, wherein participants were asked to describe each symptom they encountered either as relapsing, meaning the symptom recurs after periods of improvement or remission; persistent, meaning the symptom remains consistently present over time; fluctuating, meaning the symptom varies in intensity and frequency over time; or increasing, meaning the symptom becomes more severe or frequent over time. Across all symptoms, most reported relapsing symptoms. Statistically significant differences were determined through one-way ANOVA followed by Tukey’s multiple comparisons test to compare the mean number of reports from S1 to S3 for each symptom, highlighting the variations in the prevalence and nature of symptom reports over time (Table 5 and Supplementary Figures S1–S6).

**Table 5:** Intensity and frequency of Long COVID symptoms as described by participants.

Long COVID symptom	Mean number of reports from S1 to S3 (% of all participants *n* = 172)				*P-*value^†^	
	Relapsing	Persistent	Fluctuating	Increasing		
General symptoms						
Headache	26.74^a^	2.91^bc^	6.98^b^	0.58^c^	**<0.0001**	****
Dizziness	18.41^a^	1.16^b^	2.52^b^	0.00^b^	**<0.0001**	****
Fever	8.33^a^	0.39^b^	0.39^b^	0.00^b^	**0.0042**	**
Onset allergies	10.47^a^	6.59^ab^	5.62^b^	1.16^c^	**0.0011**	**
Tiredness or fatigue	18.60^a^	11.05^b^	7.75^bc^	1.16^c^	**0.0003**	***
Post-exertional malaise	12.40^a^	5.23^b^	6.40^ab^	0.39^b^	**0.0016**	**
Tinnitus	16.86^a^	3.29^b^	3.88^b^	1.55^b^	**<0.0001**	****
Pins-and-needles (peripheral neuropathy)	15.12^a^	3.49^b^	2.91^bc^	0.58^c^	**<0.0001**	****
Cardiopulmonary symptoms						
Difficulty breathing	14.92^a^	5.81^b^	6.40^b^	0.97^b^	**0.0024**	**
Palpitations	12.98^a^	2.13^b^	2.71^b^	0.00^b^	**0.0002**	***
Cough	14.73^a^	2.33^b^	3.88^b^	0.58^b^	**<0.0001**	****
Chest pain	13.95^a^	1.36^b^	3.10^b^	0.19^b^	**<0.0001**	****
Gastrointestinal symptoms						
Stomach pain	5.62^a^	1.16^b^	1.94^b^	1.16^b^	**<0.0001**	****
Diarrhea	7.95^a^	1.16^b^	1.94^b^	0.00^b^	**0.0020**	**
Constipation	5.04^a^	2.13^b^	0.97^bc^	0.19^c^	**<0.0001**	****
Acid reflux	15.50^a^	3.68^b^	4.07^b^	2.33^b^	**<0.0001**	****
Musculoskeletal symptoms						
Muscle pain	14.92^a^	6.59^ab^	5.62^b^	1.36^b^	**0.0078**	**
Joint pain	12.79^a^	3.49^b^	2.52^b^	0.78^b^	**0.0004**	***
Neuropsychiatric symptoms						
Brain fog	23.06^a^	9.11^b^	8.14^b^	2.91^b^	**0.0034**	**
Sleep problems	11.82^a^	8.91^a^	7.95^a^	4.07^b^	**0.0133**	*
Mood changes	13.76^a^	3.10^b^	7.17^c^	2.33^b^	**<0.0001**	****
Changes in smell	7.17^a^	6.01^a^	1.74^b^	0.97^b^	**0.0020**	**
Dermatologic symptoms						
Rashes	7.56^a^	3.49^b^	3.10^b^	0.39^c^	**<0.0001**	****
Hairfall	2.13^a^	0.78^a^	0.00^a^	0.39^a^	0.0575	ns
Women-related symptoms						
Changes in menstrual cycle	3.10^a^	2.71^a^	2.13^a^	0.39^b^	**0.0026**	**

^†^Statistically significant differences were determined through one-way ANOVA followed by Tukey’s multiple comparisons test to compare the mean number of reports from S1 to S3 for each symptom. Statistical significance is indicated by superscript letters above the means; means with the same letter are not significantly different from each other. After applying multiple corrections, the threshold for statistical significance was adjusted to *P* < 0.05 (^*^P < 0.05, ^**^P < 0.01, ^***^P < 0.001, ^****^P < 0.0001, ^ns^*P* > 0.05). Bold values indicate statistically significant *P*  values.

### 3.4 The number of Long COVID symptoms varies with demographic and disease severity during acute infection

**Figure 6:**
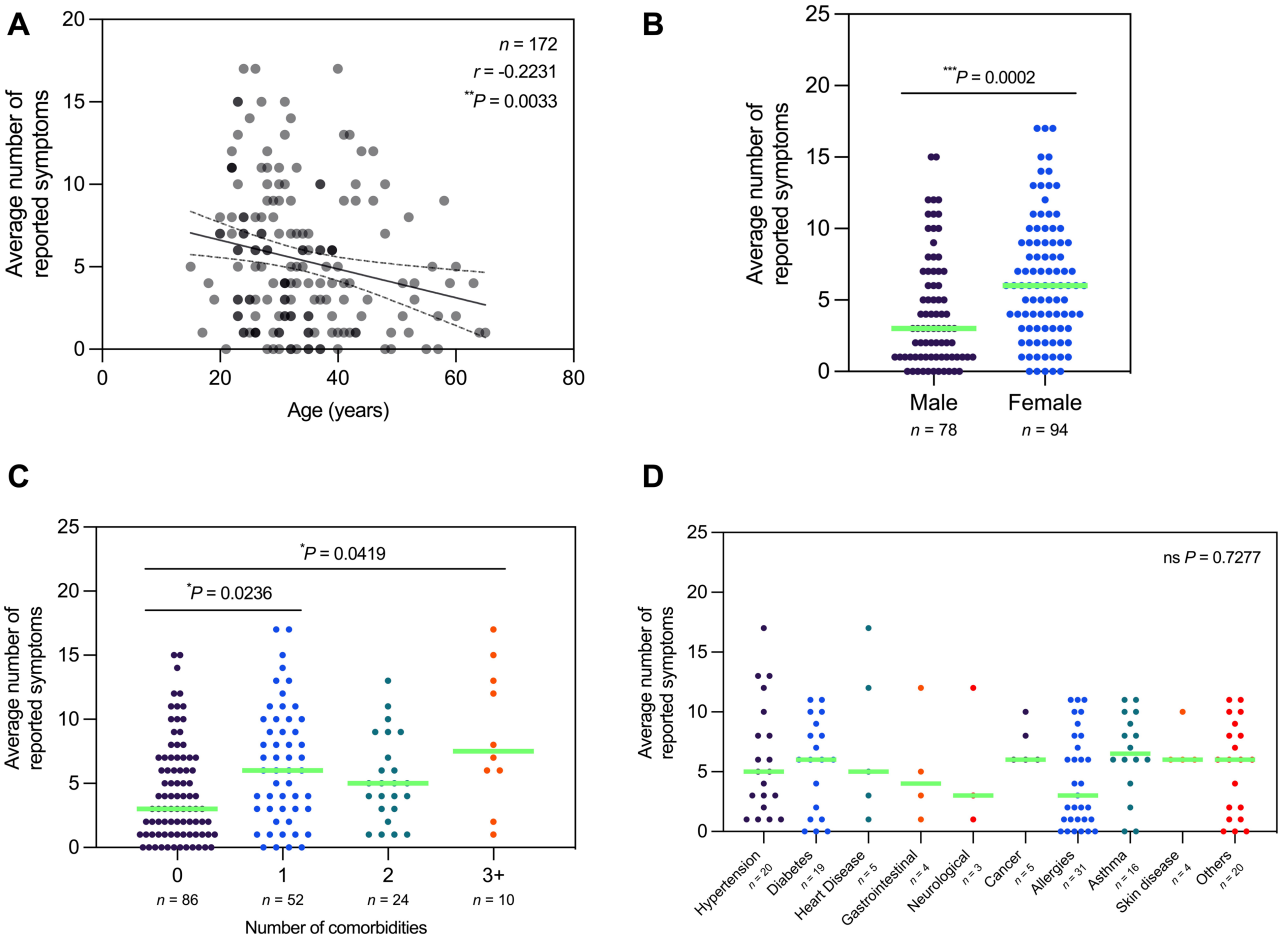
Average number of Long COVID symptoms reported varied by age, sex, and number of comorbidities. **(A)** Number of reported symptoms is negatively correlated with age (Spearman’s rank-order correlation; *r* = −0.2231; *P* = 0.0033). **(B)** Number of reported symptoms is significantly higher in females relative to males (Mann–Whitney test; *P* = 0.0002). **(C)** Number of reported symptoms is significantly higher in participants with one or three or more comorbidities relative to those with none (Kruskal–Wallis test; *P* = 0.0043). **(D)** Number of reported symptoms does not vary significantly with the presence of specific comorbidities (Kruskal–Wallis test; *P* = 0.7277). Dots represent the number of reported symptoms, with the median indicated by a neon green line. Correlation studies are depicted with a line of best fit and 95% confidence bands. Statistically significant differences were assessed using Spearman’s rank-order correlation, Mann–Whitney test, and Kruskal–Wallis test followed by Dunn’s multiple comparisons test (^*^P < 0.05, ^**^P < 0.01,^***^P < 0.001, ^ns^*P* > 0.05).

To determine if the prevalence of Long COVID varies with different variables, the average number of Long COVID symptoms from all three sessions was plotted by demographic, vaccination profile, and infection profile. In terms of age, the average number of reported symptoms exhibited a negative correlation with age, wherein those of older age reported less symptoms on average (Figure 6A). In terms of sex, females reported significantly more symptoms on average compared to males (Figure 6B). In terms of comorbidities, those with one, three, or more comorbidities reported significantly more symptoms on average compared to those with no comorbidities (Figure 6C). However, the average number of reported symptoms did not vary significantly with the presence of specific comorbidities (Figure 6D).

**Figure 7:**
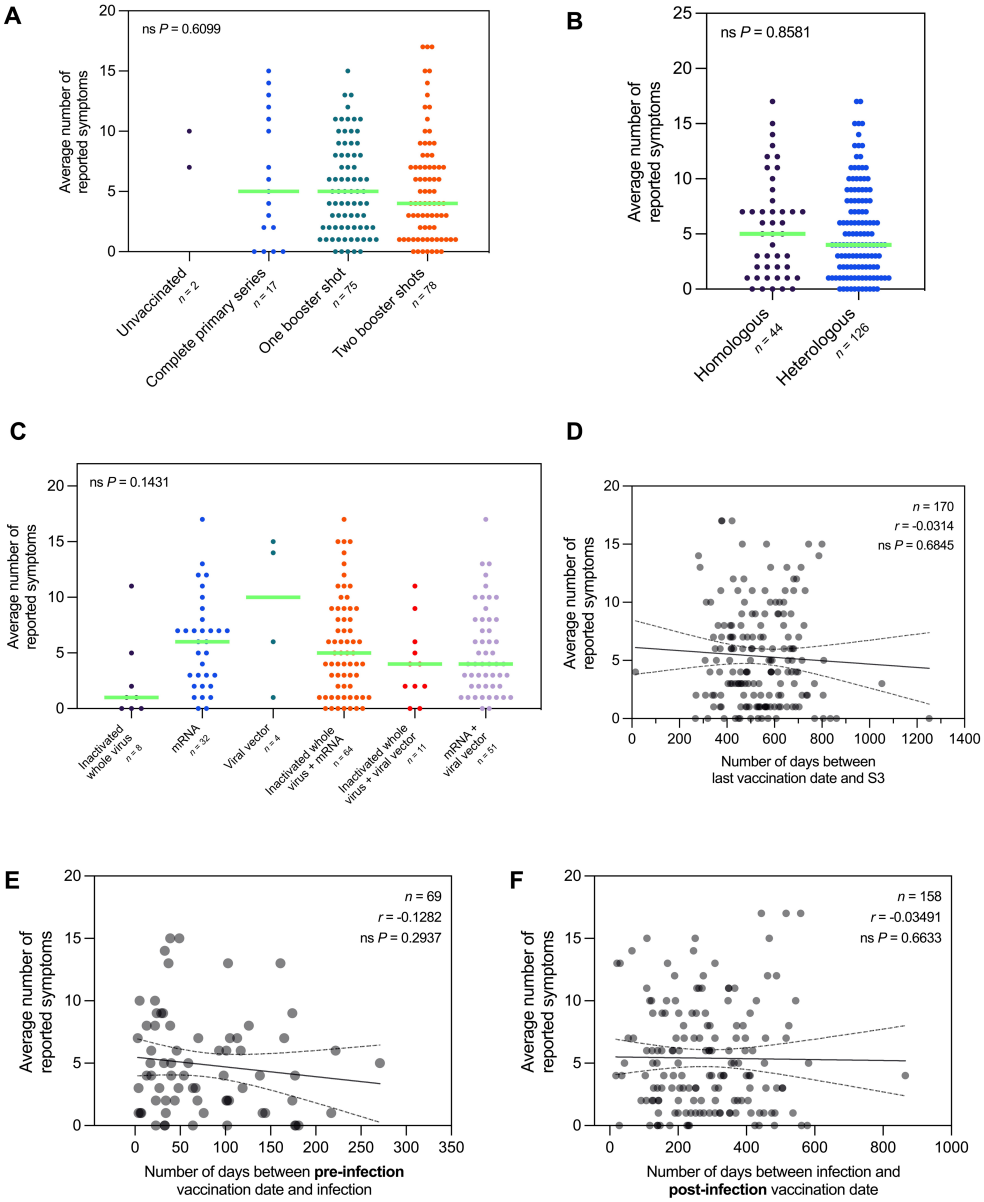
Average number of Long COVID symptoms did not vary by vaccination profile. **(A)** Number of reported symptoms does not vary significantly with vaccination status at the time of the first session (Kruskal–Wallis test; *P* = 0.6099). Likewise, the number of reported symptoms does not vary significantly with **(B)** vaccine combination taken (Mann–Whitney test; *P* = 0.8581) and **(C)** vaccine type/s taken (Kruskal–Wallis test; *P* = 0.1431). **(D)** Number of reported symptoms is not significantly correlated with the number of days between the last vaccination date and the third session (Spearman’s rank-order correlation; *r* = −0.0314, *P* = 0.6845). Likewise, the number of reported symptoms is not significantly correlated with the number of days between pre-infection vaccination date and infection **(E)** (Spearman’s rank-order correlation; *r* = −0.1282, *P* = 0.2937), and the number of days between infection and post-infection vaccination date **(F)** (Spearman’s rank-order correlation; *r* = −0.03491, *P* = 0.6633). Dots represent the number of reported symptoms, with the median indicated by a neon green line for sample sizes of three (3) or more. Statistically significant differences were assessed using Mann–Whitney test and Kruskal–Wallis test followed by Dunn’s multiple comparisons test (^*^P < 0.05, ^ns^*P* > 0.05).

The average number of reported symptoms did not vary significantly with vaccination status (Figure 7A), vaccine combination (Figure 7B), and vaccine type/s taken at the time of the first session (Figure 7C). Additionally, the average number of reported symptoms did not show a significant correlation with the number of days between the last vaccination date and the first session of the participants (Figure 7D). Furthermore, we observed that the number of reported symptoms was not significantly correlated with the number of days between infection and vaccination dates (both pre- and post-infection) (Figures 7E,F).

**Figure 8:**
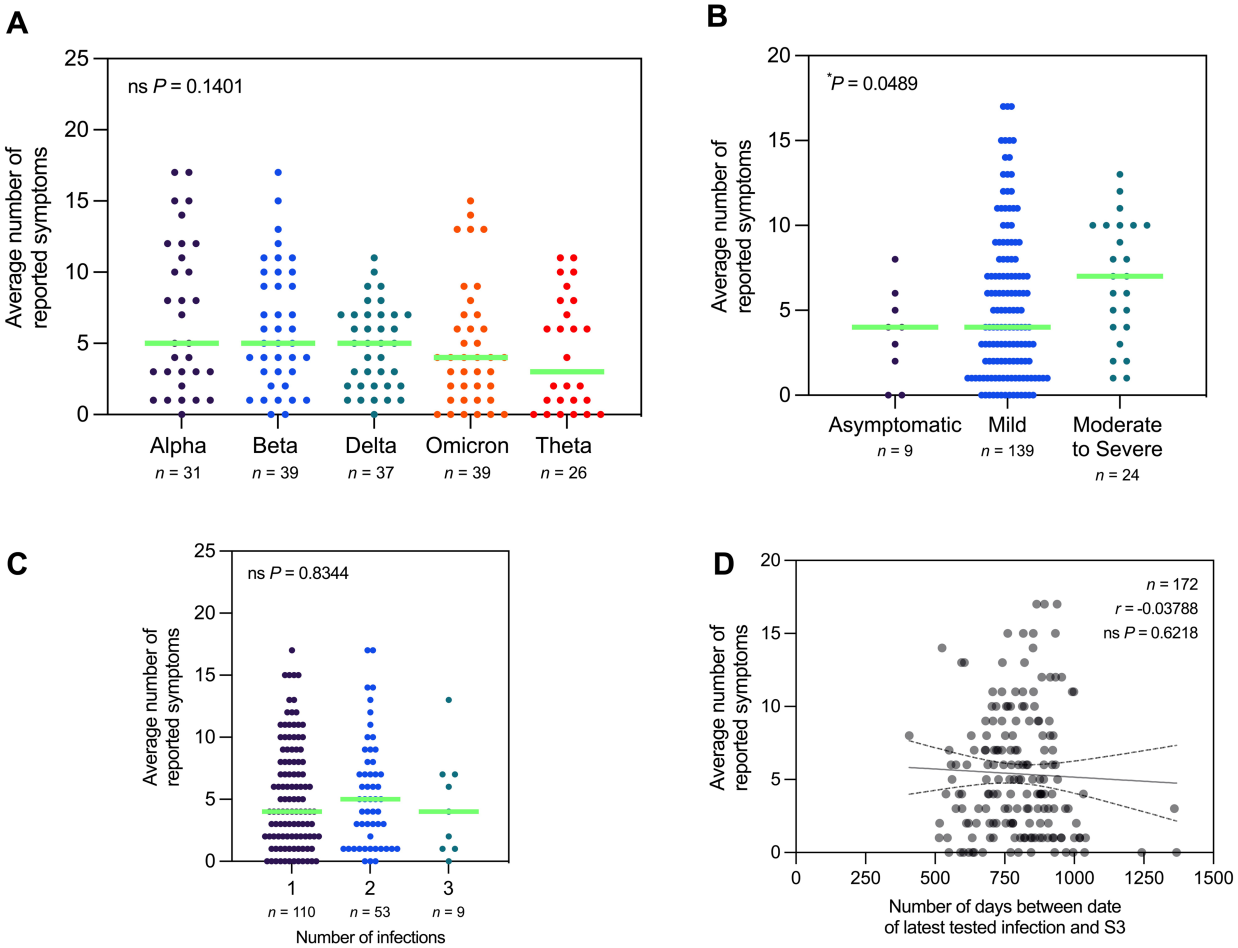
Average number of Long COVID symptoms varied by disease severity during acute infection. **(A)** Number of reported symptoms does not vary significantly with SARS-CoV-2 variant (Kruskal–Wallis test; *P* = 0.1401). **(B)** Number of reported symptoms varies significantly with disease severity during time of infection (Kruskal–Wallis test; *P* = 0.0489). **(C)** Number of reported symptoms does not vary significantly with the total number of tested infections (Kruskal–Wallis test; *P* = 0.8344). **(D)** Number of reported symptoms is not significantly correlated with the number of days between date of latest tested infection and third session (Spearman’s rank-order correlation; *r* = −0.03788, *P* = 0.6218). Dots represent the number of reported symptoms, with the median indicated by a neon green line. Correlation studies are depicted with a line of best fit and 95% confidence bands. Statistically significant differences were assessed using Spearman’s rank-order correlation and Kruskal–Wallis test followed by Dunn’s multiple comparisons test (^*^P < 0.05, ns *P* > 0.05).

Upon stratification of participants by variant, there was no significant difference in the average number of reported symptoms across all cohorts (Figure 8A). In contrast, the average number of reported symptoms varied significantly with disease severity during the time of infection. However, although one-way ANOVA indicated significant differences in the average number of reported symptoms by disease severity, the multiple comparisons test did not reveal any significant differences between specific groups (Figure 8B). In terms of number of infections, the average number of reported symptoms varied significantly with the number of tested infections before the first session and during the period of the three sessions (Figure 8C). Similarly, the average number of reported symptoms was also not significantly correlated with the number of days between date of last tested infection and S3 (Figure 8D).

## 4 Discussion

Long COVID, also known as post-acute sequelae of SARS-CoV-2 infection (PASC) is characterized by a complex and debilitating array of symptoms persisting beyond the acute phase of COVID-19. This chronic syndrome is characterized by a wide range of acquired sequela that affects many of the major organ systems in the body and can persist beyond 4 weeks from the onset of acute COVID-19 symptoms (6, 7, 25). While the cause of Long COVID remains unclear, some believe that the condition develops because of putative viral reservoirs, sustained damage from the initial infection, elevated autoantibodies in response to the initial infection, dysregulated immune response, and adverse effects of medications used (8, 26). Because Long COVID shares many features with chronic disorders brought about by other infectious agents such as myalgic encephalomyelitis or chronic fatigue syndrome (ME/CFS), this illness may involve common etiopathogenetic pathways (10, 11). Given the heterogeneous nature of this illness, understanding the symptomatology and epidemiology of Long COVID is becoming increasingly crucial as it represents a significant public health concern and poses challenges for healthcare systems worldwide. Through this retrospective-prospective study using a Philippine cohort, longitudinal analysis hopes to provide insights into the presence, progression, and persistence of Long COVID symptoms across individuals infected with different SARS-CoV-2 variants over time.

### 4.1 Long COVID symptomatology in a Philippine cohort stratified with VoC infection

Globally, although exact numbers are uncertain, studies show that as much as 60% of those infected by SARS-CoV-2 may go on to develop symptoms that can be diagnosed as Long COVID, consistent with a recent review that estimates that as much as 400 million individuals worldwide are affected by this condition (6, 27, 28). However, the prevalence of Long COVID and the number of reported cases varies globally, which could stem from underdiagnosing due to the lack of consensus on its definition or awareness of the condition, as well as underreporting since some symptoms might not have been severe enough to be noticed or reported (29). In our cohort, we found that the number of those symptomatic for Long COVID ranges between 68 and 88% of all participants, which is markedly higher than the 10–20% estimate seen in other studies (27). A pre-print meta-analysis reported as much as 80% of patients had prolonged post-infectious symptoms within a follow up period of up to 100 days post-recovery, but our study is the first in our region to evaluate symptoms after over a year post-infection (30).

The large proportion of Long COVID symptoms reported may be due in part to the nature of the follow-up method of patient interviews when recall bias could potentially influence the response of patients. The presence of a control group or a national registry for COVID-19 cases could help minimize this bias, but the identification of non-infected controls is challenging given the highly infectious nature of the virus. Although the cumulative case count of COVID-19 cases in the Philippines was roughly 4% of the population, limited availability of testing in the country underestimates the true number of cases, and as such many patients would have been exposed to the virus at the time of the study, whether through casual exposure or asymptomatic infection (31).

SARS-CoV-2 induces both direct and indirect pathology which results in dysfunction of almost all of the major organ systems in the human body (6, 32). Of the major organ systems affected by Long COVID, the nervous system and the respiratory system seem to be the most well-studied as the majority of those with Long COVID report neuropsychiatric and pulmonary symptoms such as brain fog, sleep problems, dyspnea, and fatigue (33, 34). Consistent with these findings, neuropsychiatric Long COVID symptoms represented one of the top categories of the most frequently reported symptoms across all reports in our cohort.

Of the neuropsychiatric symptoms, the majority of the participants reported brain fog, which is an umbrella term encompassing a wide range of cognitive impairments such as memory loss, confusion, mental blocks, and difficulty thinking or concentrating (35). Consistent with the definition of brain fog, individuals in our cohort who experienced intermittent brain fog noted that they experienced disorientation, periods of dissociation, memory lapses, and that they struggle with maintaining focus over extended periods, recalling words, and remembering immediate tasks (35). The heterogeneous nature of brain fog poses significant challenges to professional performance and the overall quality of life for individuals, which thus underscores the importance of investigating its etiology and exploring its manifestations across diverse populations (36). Notably, the majority of the brain fog reports came from the Alpha and Beta cohorts, which suggests differences in neurotropism across variants and how these earlier variants could increase the risk of developing brain fog and other neurocognitive impairments. Some believe that this association may be attributed to the cumulative burden of adverse psychological and social factors linked to the prolonged duration of the pandemic (6, 10).

Apart from brain fog, headache is another neurological symptom commonly reported among those who develop Long COVID. Long COVID headache can present in the form of a worsening of a pre-existing headache or in the form of an intermittent or fluctuating headache after acute infection; the latter being consistent with findings from our study (37, 38). Notably, the majority of the headache reports came from either the Alpha or Beta cohort, and headache was consistently a top reported symptom in the Omicron cohort in all three sessions, which suggests an association between specific variants and Long COVID headaches.

Alongside brain fog and headache, fatigue was one of the most frequently reported symptoms among all participants in all three sessions, similar to findings from Italy and UK cohorts wherein the majority also reported experiencing chronic fatigue post-infection (6, 10). Because COVID-19 is primarily a respiratory infection, it makes sense that long-term pulmonary abnormalities constitute a central aspect of Long COVID syndrome (10). Among the fatigue reports, the majority came from either the Alpha or Beta cohorts, similar to our findings on brain fog and fatigue, while a minority came from either the Omicron or Theta cohorts. These contrasting results, again, highlight the differences in the risk of developing Long COVID between earlier circulating variants and later circulating variants (10).

In general, we observed that most of the brain fog, headache, and fatigue reports came from either the Alpha or Beta cohorts in our study, which points to the possibility that those infected by earlier variants are at higher risk of developing these symptoms. While the reason behind the co-occurrence of brain fog, headache, and fatigue in a number of recovered cases remains unclear, these symptoms share common underlying pathophysiological mechanisms, including inflammation, dysregulated immune responses, neurotransmitter imbalances, and alterations in cerebral blood flow, which could explain why these symptoms were consistently among the top reported symptoms in all three sessions (10, 35, 39). In addition, central sensitization has also been linked to Long COVID and may explain the prevalence of these three symptoms (40). This phenomenon is characterized by CNS hypersensitivity to sensory stimuli that leads to the amplification of neurocognitive symptoms such as headache and brain fog, as well as increased fatigue due to heightened neural responses (40).

Brain fog, headache, fatigue, and the other symptoms assessed in this study were all consistently reported as relapsing or intermittent among the participants, consistent with a meta-analysis showing a decreased prevalence of symptoms 30 days after onset, a posterior increase 60 days after but with another decrease > 90 days after (41). The relapsing nature of these symptoms may explain the observed decline in the number of symptomatic cases throughout the three sessions; however, it is possible that during subsequent sessions, the symptoms simply had not recurred yet in some individuals, which thus highlights the dynamic and temporal nature of Long COVID and need for longitudinal monitoring throughout weeks, months or years after the infection (42). Alternatively, the observed decline in the number of symptomatic cases on subsequent sessions could represent improvements or resolution of symptoms during the study period; however, this assumption should be taken with caution as potential recall bias among the participants could have masked the true prevalence symptoms in the study population.

### 4.2 Variability of Long COVID manifestation, duration, and its episodic nature

Previous studies have identified age as a risk factor for the severity of acute infection and the development of Long COVID, with older individuals at higher risk of developing sequelae post-infection (43, 44). Older individuals, in particular, are more likely to have pre-existing conditions and comorbidities, which makes them more susceptible to severe disease and sequelae post-infection (6). Contrasting results were observed in our cohort, wherein age was negatively correlated with the number of reported symptoms, but such findings could be attributed to the limited sample size for older age groups. The disparity in these findings underscores the need for additional research to clarify underlying mechanisms and discern patterns of Long COVID across various age groups.

Although women seem to experience less severe complications during the acute phase of COVID-19, they seem to suffer worse long-term complications post-infection (45). In our cohort, females exhibited a higher prevalence of Long COVID symptoms compared to males on average, consistent with previous findings which illustrate how females are at higher risk of Long COVID compared to men (7, 46). Although the precise mechanism driving these distinctions remains unclear, existing data suggests that sex-related variations in hormones and immune responses may contribute to a heightened inflammatory state during the acute phase, persisting even after recovery in females (46). While in other studies, no gender association was observed, these contrasting results may be due to differences in ethnicity and socio-economic status (46).

Pre-existing conditions and comorbidities correlate with the severity at the acute stage and are also risk factors for developing Long COVID (6). A previous study found that patients with three or more comorbidities are at a two-fold risk of not returning to the basal health status (47). We observe similar findings in our study wherein the prevalence of Long COVID is higher in those with comorbidities than those without, particularly in those with one, three, or more comorbidities. While we were only able to assess the number of reported symptoms based on the presence and number of comorbidities, it is worth noting that certain comorbidities (e.g., Type 2 diabetes, obesity, mental illness) may make an individual more predisposed to Long COVID than others (6, 47, 48).

It is well-established that vaccines reduce the severity of acute SARS-CoV-2 infection. However, whether or not this confers protection against the development of Long COVID has yet to be established. While some studies report a reduction in Long COVID symptoms and symptom severity after one or two doses of vaccine, others report no change or even worsening of symptoms after vaccination. Various studies suggest that vaccination generally tends to lessen the severity and frequency of these symptoms, highlighting the potential protective effect of vaccines against the development or persistence of Long COVID (49–52). Although the findings of the present study show that there is no significant difference in the number of reported symptoms between the different vaccination groups, it should be emphasized that no statistical analysis could be reliably applied to the unvaccinated group due to the limited number of participants in the cohort (*n* = 2). Majority of patients have already received at least one type of vaccine at the time of recruitment. An observable trend toward less symptoms with increasing number of vaccine doses can be inferred from the data suggesting the potential effect of antigen diversity in heterologous dosing strategies and inactivated whole virus preparations. This is consistent with recent systematic reviews supporting the idea of a protective effect of vaccines on Long COVID, thus underscoring the importance of comparative studies to help clarify this effect (49, 53).

Another curious observation would be the timing of vaccination to proliferation of variants. As vaccines had yet to be distributed during the peak periods of Alpha and Beta variant proliferation, greater vaccine exposure and uptake during the Omicron surge may explain differences noted in symptoms reported by patients albeit not statistically significant. Theta, which proliferated during the Delta surge, was noted to have less reported symptoms despite being in the same vaccination time frame as Delta. This may suggest that despite displaying spike protein mutations consistent with Beta, which could confer for it heightened immune evasion capabilities, it could be surmised that Theta exhibited a milder clinical phenotype that translates to a similarly lower incidence of Long COVID symptoms (54). However, a small Theta population precludes any definitive assessment regarding the severity of the variant, but still provides some insight to the role of vaccination status on COVID-19 severity and Long COVID sequelae.

In terms of vaccine type, the risk of developing Long COVID does not vary across different vaccine types and mechanisms, consistent with the results from our study (55, 56). However, it is worth noting that the assumptions made are limited to the vaccine brands examined in this study and the small sample size of those who took a homologous vaccine combination. Although vaccination status, vaccine combination, and vaccine types seem to have no appreciable outcome on Long COVID development in our Philippine cohort, vaccination may emerge as a potential therapeutic for those with Long COVID by resetting a dysregulated immune response after acute infection or by eradicating residual viral reservoir (49). We also examined the timing of vaccination relative to onset of infection to understand how this might affect the risk of developing Long COVID. The lack of significant correlation between the number of days between infection and vaccination date, either pre- or post-infection, indicates that the timing of vaccination, whether before or after infection, did not significantly influence the development of symptoms in our cohort. Taken together, our results suggest that vaccination status at the time of infection did not influence the manifestation of Long COVID symptoms. It remains to be seen, however, whether vaccination status will have any effect on Long COVID symptom severity.

Many studies have described the sequelae of acute SARS-CoV-2 infection, however, the association between changes in the SARS-CoV-2 genetic code and the development of Long COVID has been poorly understood (57). Because of fitness-enhancing mutations in the viral genome, different variants have varying degrees of transmissibility and virulence, which affects both acute infection and Long COVID (6). In general, patients infected with an earlier variant (e.g., Alpha, Beta, and Theta) tend to be at higher risk of developing Long COVID than those infected with a subsequent variant (e.g., Omicron or Delta); however, this assumption should be considered under the potential effect of reinfections and vaccines (42). For instance, one study found that cases attributed to the Omicron variant had lower odds of developing long-term cardiopulmonary symptoms compared to those attributed to the Delta variant (57). In another study, they found that those infected with Delta and Omicron reported less severe olfactory dysfunction than those infected with the wild-type strain (10). In this study, however, symptom prevalence did not vary significantly upon stratification of participants by variant and the number of reported symptoms did not vary significantly across those previously infected with different SARS-CoV-2 variants, contrary to findings that suggest that certain phenotypic presentations of Long COVID may be associated with previous exposure to specific SARS-CoV-2 variants (58, 59). Future studies with larger sample sizes and longitudinal designs should further investigate the variability of Long COVID symptoms in relation to different SARS-CoV-2 variants that circulated in the Philippines. This is of particular importance given the distinct demographic profile of the Philippines, characterized by a unique population of mobile workers, including over 100,000 Overseas Filipino Workers (OFWs) who returned home during the pandemic, heightening the potential for exposure to diverse viral strains and other health risks (60).

While differences in virulence and transmissibility of SARS-CoV-2 variants may account for the development of Long COVID, reports show that disease severity during acute infection and subsequent reinfections may also be associated with the development and severity of Long COVID. A previous study shows that those with severe disease during acute infection reported a higher number of Long COVID symptoms, consistent with our findings that those with moderate to severe disease reported a higher number of symptoms compared to those with mild disease (61). Moderate to severe cases of COVID-19 are often associated with higher viral loads and greater tissue damage throughout the body, both of which are known to contribute to the development of Long COVID (48). In addition, prolonged hospital stays, physical deconditioning, and complications associated with critical illness can further exacerbate the risk of long-term physical, cognitive, and psychological impairments. Although the number of studies examining the relationship between Long COVID and multiple reinfections is limited, the consensus at present is that reinfection further increases the risk of Long COVID sequelae in the acute and post-acute phase, contrary to what we observed in our cohort (55, 62). While both symptomatic and asymptomatic SARS-CoV-2 reinfections may result in Long COVID, the risk of developing Long COVID was found to be significantly lower in asymptomatic individuals (63).

Another important aspect to consider is the variability in Long COVID severity, which can range from mild complaints to life-changing debilitation (64). In this study, we assessed Long COVID severity by counting the number of reported symptoms. However, this approach does not fully capture the impact of Long COVID on the quality of life. A follow-up study involving quality of life surveys and patient-administered disability rating scales can help quantify the long-term sequelae and impact of Long COVID on this cohort. This is particularly important in the context of the Philippines where the impression of medical practitioners on the severity of Long COVID symptoms has yet to be studied despite a general assumption that most reported cases of Long COVID are mild and not as debilitating as reported elsewhere (65). Such non-chalance may also be due to a lack of awareness, highlighting the need for a multifaceted approach in assessing Long COVID that captures both quantitative and qualitative symptoms.

## 5 Summary

Long COVID presents as a long-term complication of COVID characterized by a highly heterogeneous set of debilitating symptoms. In this retrospective-prospective study using a Philippine cohort, we were able to identify the presence, intensity, and number of Long COVID symptoms across the dominant SARS-CoV-2 variants circulating in the country. We found that a large proportion of participants who were infected from 2021 to 2022 reported intermittent fatigue, headache, and brain fog even after more than a year post-infection consistent with other studies. The findings of our study provide a valuable foundation for developing interventions and treatment strategies to help address the challenge of rehabilitating patients facing a disease with a myriad of clinical presentations. Furthermore, it highlights the need for long-term monitoring of Long COVID and its impact on human health and the need for our health systems to adopt policy response strategies. To our knowledge, this study is the first to provide insights into post-COVID sequelae in a Philippine cohort and the possible risk factors that contribute to the prevalence of this chronic syndrome.

## Data Availability

The original contributions presented in the study are included in the article/Supplementary material, further inquiries can be directed to the corresponding author.
