# Supplementary material for: Long-term COVID-19 sequelae by Theta and SARS-CoV-2 variants in a Philippine cohort
Source: Front Med (Lausanne). 2024 Oct 2;11:1455729. doi: 10.3389/fmed.2024.1455729 (PMC11483863; doi:10.3389/fmed.2024.1455729)
Supplement: Supplementary file 1 [file Data_Sheet_1.PDF]

Supplementary Material

1 Supplementary Figures and Tables

1.1 Supplementary Figures

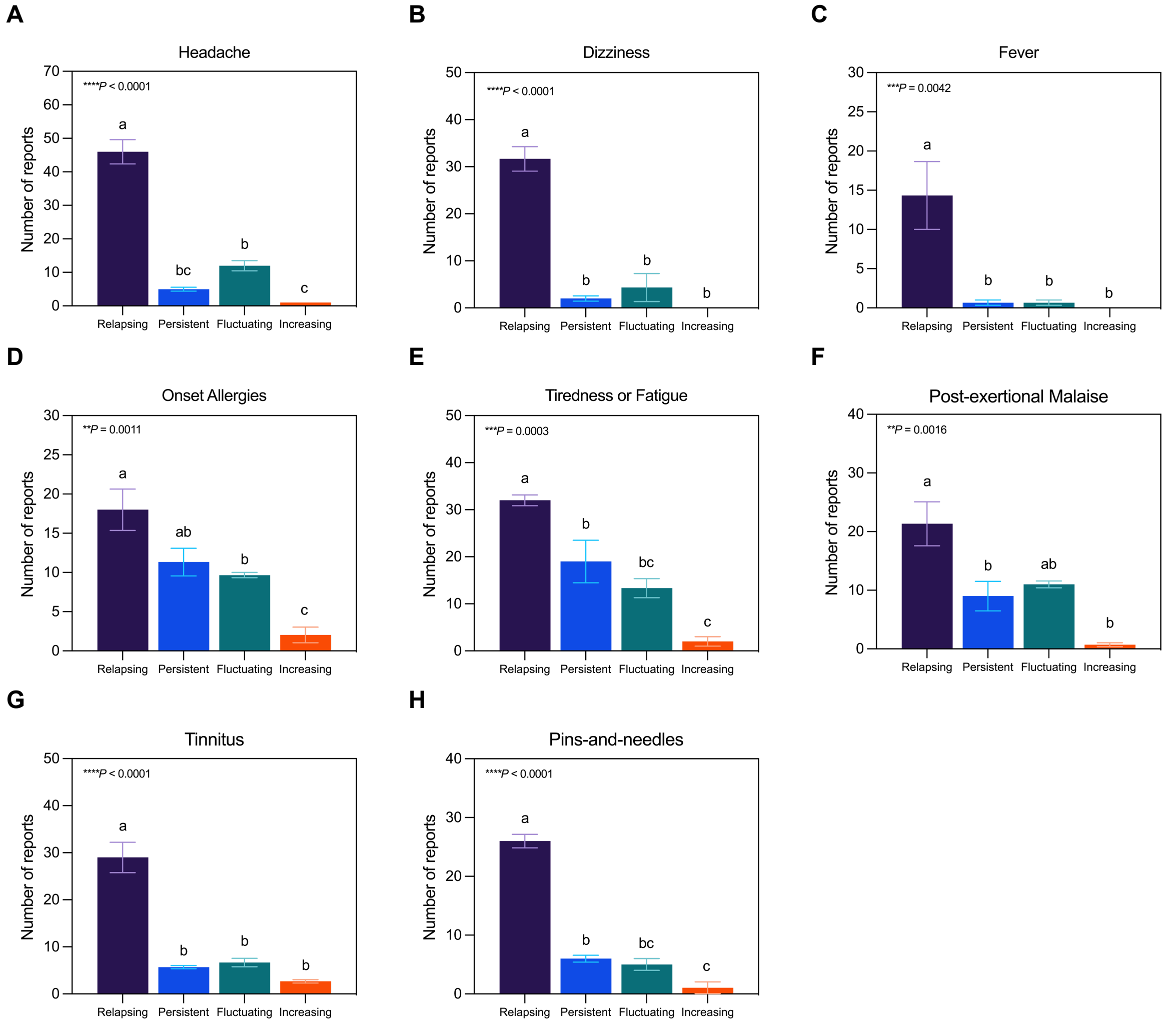

Figure 1. General Long COVID symptoms were mostly described as relapsing in all three sessions. The number of **(A)** headache (one-way ANOVA; F(3, 8) = 107.4, $P < 0.0001$), **(B)** dizziness (one-way ANOVA; F(3, 8) = 55.77, $P < 0.0001$), **(C)** fever (one-way ANOVA; F(3, 8) = 10.17, $P = 0.0042$), **(D)** onset allergy (one-way ANOVA; F(3, 8) = 15.40, $P = 0.0011$), **(E)** fatigue (one-way ANOVA; F(3, 8) = 23.24, $P = 0.0003$), **(F)** post-exertional malaise (one-way ANOVA; F(3, 8) = 13.28, $P = 0.0016$), **(G)** tinnitus (one-way ANOVA; F(3, 8) = 51.84, $P < 0.0001$), and **(H)** pins-and-needles (one-way ANOVA; F(3, 8) = 137.1, $P < 0.0001$) reports varies significantly across different intensity and frequency categories. Bars represent mean ± SEM with statistical significance indicated by letters above the means in one-way ANOVA followed by Tukey's multiple comparisons test ($P < 0.05$; means with the same letter are not significantly different).

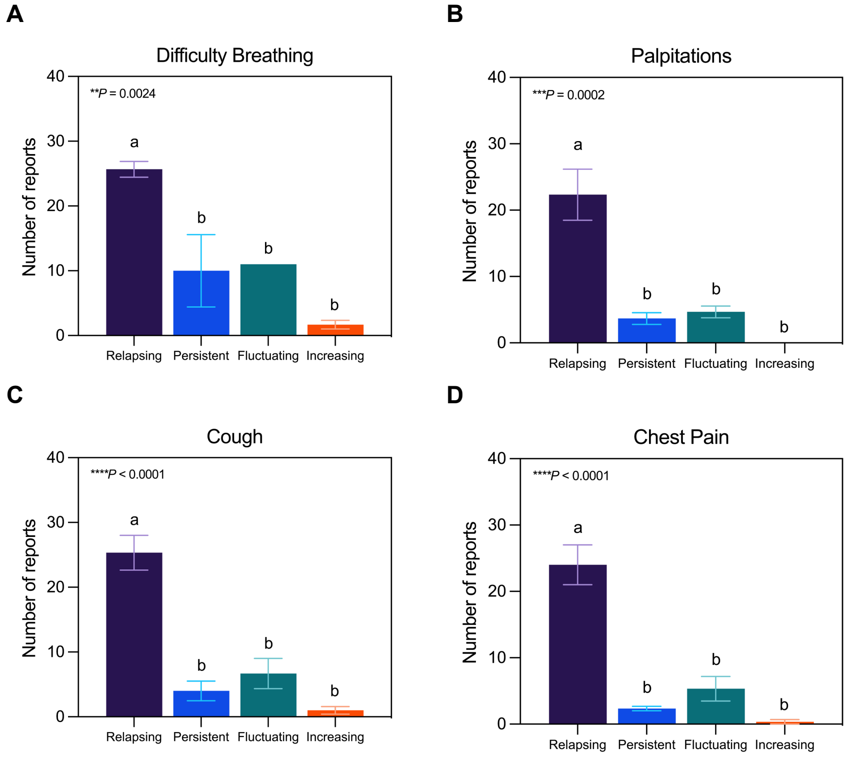

Figure 2. **Cardiopulmonary Long COVID symptoms were mostly described as relapsing in all three sessions**. The number of **(A)** dyspnea (one-way ANOVA; $F(3, 8) = 12.19$, $P = 0.0024$), **(B)** palpitation (one-way ANOVA; $F(3, 8) = 24.40$, $P = 0.0002$), **(C)** cough (one-way ANOVA; $F(3, 8) = 31.62$, $P < 0.0001$), and **(D)** chest pain (one-way ANOVA; $F(3, 8) = 37.26$, $P < 0.0001$) reports varies significantly across different intensity and frequency categories. Bars represent mean ± SEM with statistical significance indicated by letters above the means in one-way ANOVA followed by Tukey's multiple comparisons test ($P < 0.05$; means with the same letter are not significantly different).

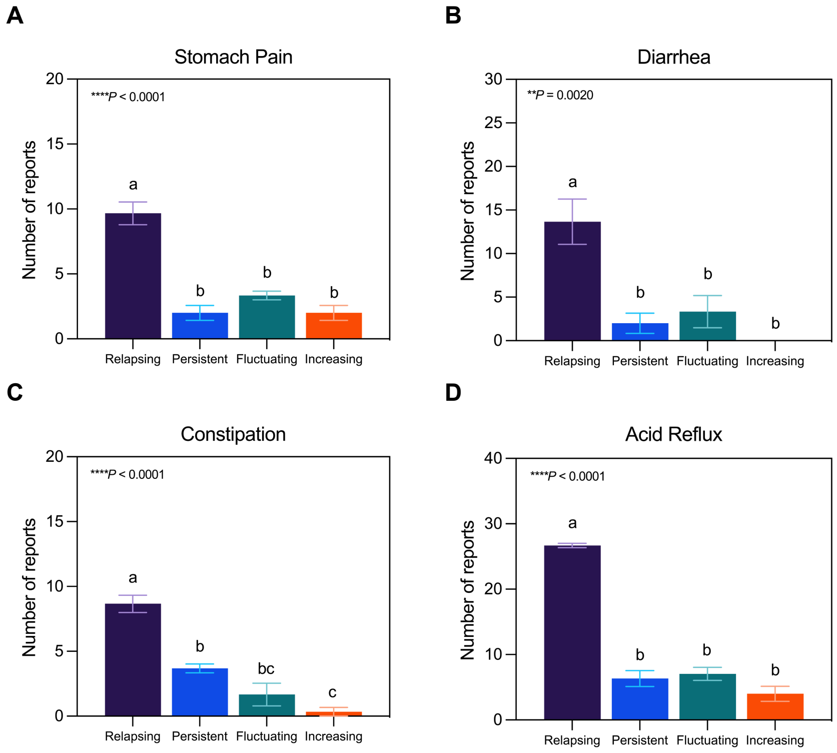

Figure 3. **Gastrointestinal Long COVID symptoms were mostly described as relapsing in all three sessions**. The number of **(A)** stomach pain (one-way ANOVA; $F(3, 8) = 34.55$, $P < 0.0001$), **(B)** diarrhea (one-way ANOVA; $F(3, 8) = 12.88$, $P = 0.0020$), **(C)** constipation (one-way ANOVA; $F(3, 8) = 37.00$, $P < 0.0001$), and **(D)** acid reflux (one-way ANOVA; $F(3, 8) = 113.9$, $P < 0.0001$) reports varies significantly across different intensity and frequency categories. Bars represent mean ± SEM with statistical significance indicated by letters above the means in one-way ANOVA followed by Tukey's multiple comparisons test ($P < 0.05$; means with the same letter are not significantly different).

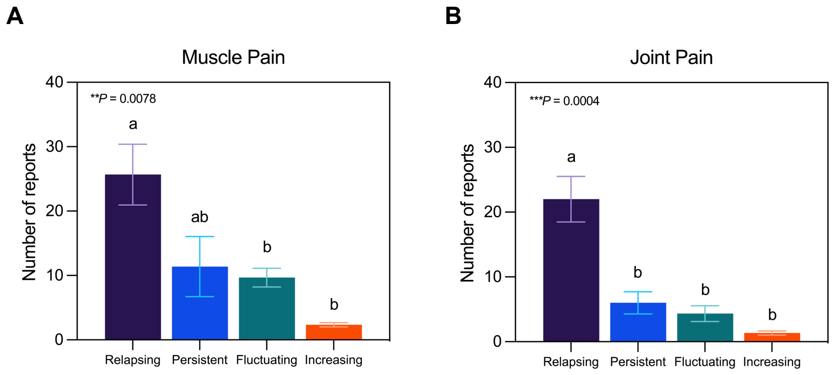

Figure 4. **Musculoskeletal Long COVID symptoms were mostly described as relapsing in all three sessions**. The number of **(A)** muscle pain (one-way ANOVA; F(3, 8) = 8.266, *P* = 0.0078) and **(B)** joint pain (one-way ANOVA; F(3, 8) = 20.30, *P* = 0.0004) reports varies significantly across different intensity and frequency categories. Bars represent mean ± SEM with statistical significance indicated by letters above the means in one-way ANOVA followed by Tukey's multiple comparisons test ($P < 0.05$; means with the same letter are not significantly different).

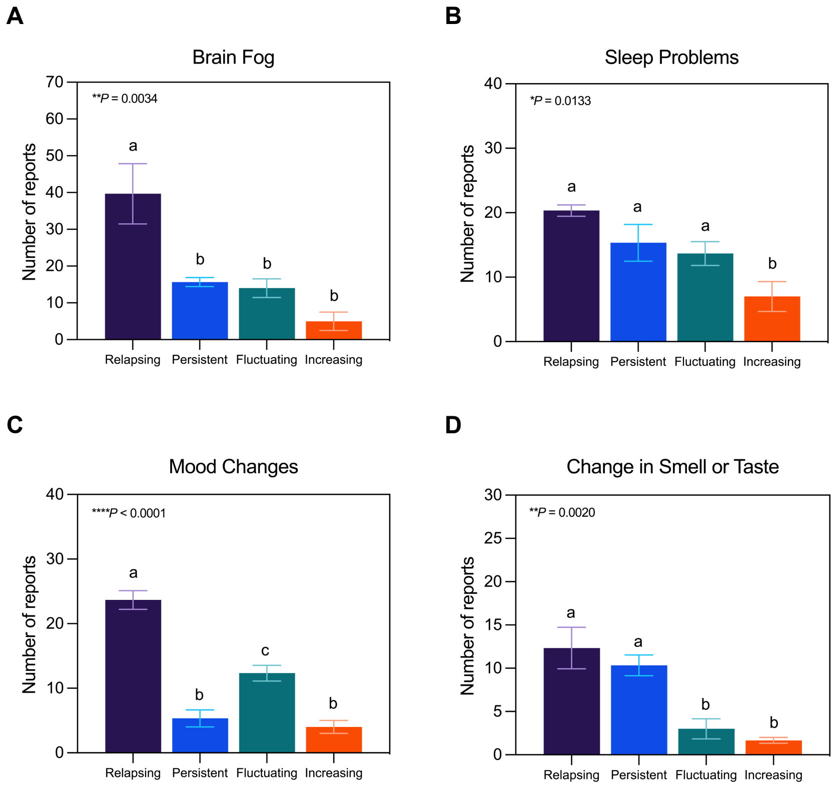

Figure 5. **Neuropsychiatric Long COVID symptoms were mostly described as relapsing in all three sessions**. The number of **(A)** brain fog (one-way ANOVA; $F(3, 8) = 10.85$, $P = 0.0034$), **(B)** sleep problem (one-way ANOVA; $F(3, 8) = 6.866$, $P = 0.0133$), **(C)** mood change (one-way ANOVA; $F(3, 8) = 51.13$, $P < 0.0001$), and **(D)** changes in smell or taste (one-way ANOVA; $F(3, 8) = 12.91$, $P = 0.0020$) reports varies significantly across different intensity and frequency categories. Bars represent mean ± SEM with statistical significance indicated by letters above the means in one-way ANOVA followed by Tukey's multiple comparisons test ($P < 0.05$; means with the same letter are not significantly different).

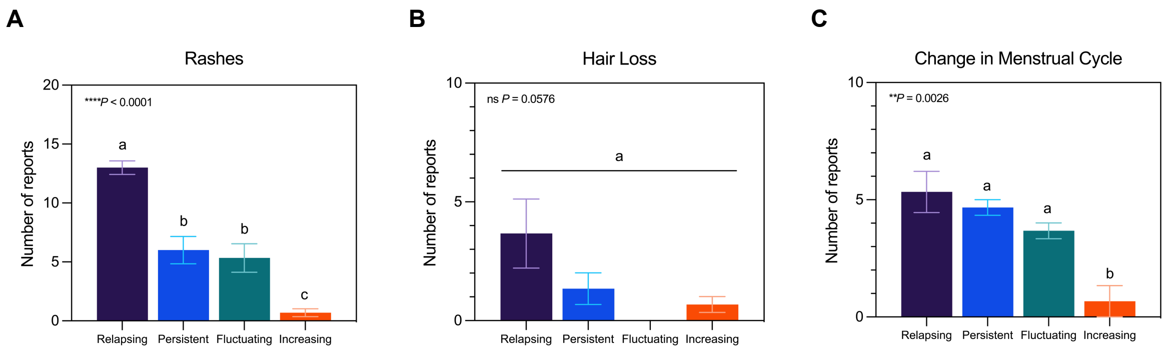

Figure 6. **Dermatologic and women-related Long COVID symptoms were mostly described as relapsing in all three sessions**. **(A)** The number of rashes reports varies significantly across different intensity and frequency categories (one-way ANOVA; $F(3, 8) = 32.13$, $P < 0.0001$). **(B)** The number of hair loss reports does not vary significantly across different intensity and frequency categories (one-way ANOVA; $F(3, 8) = 3.819$, $P = 0.0576$). **(C)** The number of changes in menstrual cycle reports varies significantly across different intensity and frequency categories (one-way ANOVA; $F(3, 8) = 11.77$, $P = 0.0026$). Bars represent mean ± SEM with statistical significance indicated by letters above the means in one-way ANOVA followed by Tukey's multiple comparisons test ($P < 0.05$; means with the same letter are not significantly different).
